# Ubiquitin Carboxyl Terminal Hydrolyase L1 -Suppressed Autophagic Degradation of p21^WAF1/Cip1^ as a Novel Feedback Mechanism in the Control of Cardiac Fibroblast Proliferation

**DOI:** 10.1371/journal.pone.0094658

**Published:** 2014-04-14

**Authors:** Xiaoming Zhang, Linlin Guo, Ting Niu, Lei Shao, Huanjie Li, Weiwei Wu, Wenjuan Wang, Linmao Lv, Qingyun Qin, Fang Wang, Dongqi Tang, Xing Li Wang, Taixing Cui

**Affiliations:** 1 Shandong University Qilu Hospital Research Center for Cell Therapy, Key Laboratory of Cardiovascular Remodeling and Function Research, Qilu Hospital of Shandong University, Jinan, Shandong, China; 2 Department of Pathophysiology, Shandong University School of Medicine, Jinan, Shandong, China; 3 Department of Cell Biology and Anatomy, University of South Carolina School of Medicine, Columbia, South Carolina, United States of America; Mayo Clinic, United States of America

## Abstract

**Aims:**

Deubiquitinating enzymes (DUBs) appear to be critical regulators of a multitude of processes such as proliferation, apoptosis, differentiation, and inflammation; however, the potential roles of DUBs in the heart remain to be determined. This study was aimed to explore the role of a DUB, ubiquitin carboxyl terminal hydrolyase L1 (UCH-L1) in maladaptive cardiac remodeling and dysfunction.

**Methods and Results:**

Maladaptive cardiac remodeling and dysfunction were induced in mice by transverse aortic constriction (TAC). UCH-L1 expression was transiently increased and then declined near to the basal level while impairment of cardiac function proceeded. The upregulation of UCH-L1 was observed in cardiac myocytes and fibroblasts. In primary culture of cardiac fibroblasts, UCH-L1 was upregulated by platelet-derived growth factor (PDGF)-BB and PDGF-DD. Adenoviral overexpession of UCH-L1 inhibited the PDGF-induced cardiac fibroblast proliferation without affecting the activation of mitogen activated protein kinases (MAPKs), Akt, and signal transducers and activators of transcription 3 (STAT3). Further signaling dissection revealed that PDGF-BB posttranscriptional upregulated p21^WAF1/Cip1^ protein expression, which was inhibited by rapamycin, an activator of autophagy via suppressing mammalian target of rapamycin (mTOR), rather than MG132, a proteasome inhibitor. Overexpression of UCH-L1 enhanced PDGF-BB-induced mTOR phosphorylation and upregulation of p21^WAF1/Cip1^ protein expression while suppressed autophagic flux in cardiac fibroblasts.

**Conclusion:**

UCH-L1 facilitates PDGF-BB-induced suppression of autophagic degradation of p21^WAF1/Cip1^ proteins in cardiac fibroblasts, which may serve as a novel negative feedback mechanism in the control of cardiac fibroblast proliferation contributing to cardiac fibrosis and dysfunction.

## Introduction

Since ubiquitin (Ub) was discovered in the early 1970's, the ubiquitination proteasome system (UPS) which consists of ubiquitin-activating enzymes (E1s), ubiquitin-conjugating enzymes (E2s), ubiquitin ligases (E3s), proteasomes, and deubiquitinating enzymes (DUBs) has emerged as a critical regulator in virtually all aspects of cell biology [1]–[4]. The process of ubiquitination is catalyzed by the sequential action of E1, E2, and E3 and DUBs mediate the removal and processing of ubiquitin. There are eight E1s, a dozen different types of E2s, and hundreds of E3s in human. In addition, approximately 100 functional DUBs are coded in human genome.

Recently, rapidly growing evidence has revealed that ubiquitin, E1s, several E2s and E3s play an important role in the regulation of cardiac homeostasis and dysfunction [1], [5]–[9]. In contrast, only one DUB, A20/ tumor necrosis factor alpha induced protein 3 (TNFAIP3), has been extensively studies in the heart. While A20 appears to be a key negative regulator of maladaptive cardiac remodeling and dysfunction induced by pressure overload or myocardial infarction [10], [11], up-regulations of a few other DUBs including ubiquitin-specific protease 5 (USP5), USP20, and ubiquitin carboxyl terminal hydrolyase L1 (UCH-L1) in failed hearts have also been observed [5]. However, the pathophysiological relevance of these DUBs in the heart remains unknown.

In the present study, we explored the role of UCH-L1 in the heart. Our results uncovered for the first time that UCH-L1 facilitates autophagic degradation of p21^WAF1/Cip1^ to suppress proliferation in cardiac fibroblasts, potentially acting as a novel feedback mechanism in the regulation of maladaptive cardiac remodeling and dysfunction.

## Materials and Methods

### Animals

Male C57BL/6J mice were purchased from Shanghai Slac Laboratory Animal Co., Ltd. The mice at age of 8 weeks were anesthetized with a single intraperitoneal (IP) injection of pentobarbital sodium (50 mg/kg) and the depth of anesthesia was monitored by assessing palpebral reflex, toe pinch, respirations, and general response to touch. The anesthetized mice were subjected to sham or transverse aortic constriction (TAC) operations as described previously [12]. At endpoints of experiments, mice were euthanized by overdose intraperitoneal injection of pentobarbital sodium (100 mg/kg) for harvesting tissues. All of the animal procedures were conducted in accordance with the NIH *Guide for Care and Use of Laboratory Animals* and were approved by the Institutional Animal Care and Use Committee at Shandong University and University of South Carolina, USA.

### Immunohistochemistry

Paraffin sections of the heart were prepared and stained with a rabbit anti-UCH-L1 polyclonal antibody (AB1761, Millipore) as previously described [12]. Images were acquired using a microscope (Nikon Eclipse 80i; Nikon Inc., Melville, NY). Immunofluorescent staining was performed according to a standard protocol provided by Santa Cruz Biotechnology, Inc. Ki67, a biomarker of cell proliferation, was stained with a rabbit anti-Ki67 antibody (ab15580, Abcam). Cardiac fibroblasts were stained with a mouse anti-Vimentin antibody (V2258, Sigma-Aldrich). Nuclei were labeled with 4′,6-diamidino-2- phenylindole (DAPI; D9542, Sigma-Aldrich).

### Cell Culture and Adenovirus Infection

Rat neonatal cardiac fibroblasts were isolated and cultured as previously described [12]. Briefly, the hearts from 2- to 3-day old Wistar rats were finely minced and digested with type II collagenase (120 units/ml; Worthington Biochemical Corp., Lakewood, NJ). Dispersed cells were placed in culture flask for 90 minutes at 37°C in a CO_2_ incubator. During this time, only the fibroblasts became attached to the culture flask. The fibroblasts were cultured in high glucose DMEM supplemented with 10% fetal bovine serum (FBS) (Gibco), penicillin (100 U/ml), and streptomycin (100 mg/ml). In addition, adult cardiac fibroblasts were isolated from male New Zealand (NZ) while rabbits at age of 6 months. The rabbits were euthanized by overdose intraperitoneal injection of pentobarbital sodium and then hearts were excised, minced, and washed in phosphate-buffered saline (PBS). The tissue was digested at 37°C with a mixture of trypsin (0.125%, Invitrogen) and type II collagenase (100 units/ml; Worthington Biochemical Corp.) for 10 min. Isolated cells were pelleted at the end of several 10-min digestion periods, plated on culture flask in DMEM containing 20% FBS, and incubated for 1 h at 37°C in a CO_2_ incubator. Thereafter, the unattached cells were discarded and attached cells were grown in DMEM with 10% FBS, penicillin (100 U/ml), and streptomycin (100 mg/ml). The purity of the fibroblasts was determined by the staining of a fibroblast marker vimentin using anti-Vimentin antibody (V2258, Sigma-Aldrich). Over 95% of the cultured cells were vimentin positive. Cardiac fibroblasts at passage 2 or 3 were used for the experiments.

Sub-confluent (70–80%) rat neonatal cardiac fibroblasts were infected with adenovirus of control (Ad-GFP) or human UCH-L1 tagged with GFP (Ad-hUCH-L1) (Invitrogen) in serum free DMEM for 48 h and followed with various treatments as indicated. Adenoviral over-expression of hUCH-L1 (0–100 pfu/cell) resulted in dose-dependent increases in UCH-L1 protein expression (Figure S1), without any apparent cytotoxic effects in cardiac fibroblasts (data not shown). Accordingly, we used adenoviral over-expression of UCH-L1 at a dose of 50 pfu/cell in the following experiments.

### CCK-8 Assay

Rat cardiac fibroblasts (passage 2) were seeded in 96-well plate with 5000 cells/well, and were cultured in serum free DMEM for 24 h to induce a quiescent status. Then, cells were stimulated with or without PDGF-AA (50 ng/ml, P3076, Sigma-Aldrich), PDGF-BB (20 ng/ml, Sigma-Aldrich), PDGF-CC (50 ng/ml, SRP3139, Sigma-Aldrich), or PDGF-DD (50 ng/ml, 1159-SB, R&D) for 48 hours. Cell proliferation was assessed by a Cell Counting Kit-8 (CCK-8, Dojindo Molecular Technologies, Gaithersburg, MD). Briefly, after stimulation, the CCK-8 solution was added to the culture medium, and the cultures were incubated for 1 hour at 37°C in humidified 95% air and 5% CO2. The absorbance was measured at 450 nm using a Microplate Reader (Bio-Rad, Hercules, CA).

### Cell Counting

Rat cardiac fibroblasts (passage 2) were seeded in 6-well plates and cultured in DMEM supplemented with 10% FBS, penicillin (100 U/ml) and streptomycin (100 mg/ml). When cells reached to 50–60% confluent, cells were infected with adenovirus of UCH-L1 or GFP (50 pfu/cell) in serum free DMEM for 48 h, and then stimulated with PDGF-BB (20 ng/ml, Sigma-Aldrich) for additional 48 hours. Cells were trypsinized and counted using TC10 Automated Cell Counter (Bio-Rad, Hercules, CA).

### Reverse Transcription-Polymerase Chain Reaction (RT-PCR) and Quantitative Real Time PCR (qPCR)

Total RNA from the left ventricles or cardiac fibroblasts was extracted using TRIzol (15596-018, Invitrogen) following the standard protocol, and reverse transcription reactions (RevertAid First Strand cDNA Synthesis Kit, K1622, Fermentas) were performed with 2 µg of total RNA. Quantitative real time PCR (Q-PCR) was carried out using the Bio-Rad CFX96 Real-Time System (Bio-Rad, Hercules, CA). Expression levels of target genes were normalized by concurrent measurement of glyceraldehydes-3-phosphate dehydrogenase (GAPDH) or β-actin mRNA levels as described previously [12]. Primers that were used for qPCR are provided in supplementary data.

### Western Blot Analysis

Cell lysate preparation and Western blot were performed as previously described [13]. The primary antibodies of anti-UCH-L1 (AB1761), anti-UCH-L1 (ab8189), and anti-β-actin (TA-09) were purchased from Millipore, Abcam, and Zhongshan in China, respectively. Anti-phospho (Thr202/Tyr204)-ERK1/2 (#9101), anti-ERK1/2 (#4695), anti-phospho (Thr180/Tyr182)-p38 (#4631), anti-p38 (#4631), anti-phospho (Thr183/Tyr185)-JNK (#4668), anti-JNK (#9258), anti-phospho (Ser473)-Akt (#4058), anti-Akt (#9272), anti-phospho (Ser727)-Stat3 (#9134), anti-Stat3 (#4904), anti-phospho (Ser21/9)-GSK-3α/β (#5676), anti-GSK-3α/β (#5676), anti-phospho (Ser2448)-mTOR (#5536), anti-mTOR (#2983), and anti-LC3B (#2775) were purchased from Cell Signaling Technology. Anti-p21 (sc-397), anti-p27 (sc-528), anti-Cdk2 (sc-163), anti-Cdk4 (sc-260), anti-cyclin D1 (sc-718), anti-cyclin E (sc-481), and anti-GAPDH (sc-25778) were purchased from Santa Cruz. Bands were visualized with Immobilon Western Chemiluminescent HRP substrate (WBKLS0100, Millipore). Densitometric analysis was performed using AlphaView SA image software (Cell Biosciences).

### Immunoprecipitation

Cell lysate preparation and Western blot were performed as previously described [13]. Briefly, the precleared lysates with 40 µl protein A+G Agrose (P2012, Beyotime, China) were incubated with 1 µg rabbit anti-p21 antibody (sc-397, Santa Cruz) or 1 µg normal rabbit IgG with constant rotating at 4°C overnight, and then were incubated with protein A+G Agrose for 2 hours. The immunoprecipitates were washed 5 times with cell lysis buffer, then, the bound proteins were denatured with 40 µl 2×sample buffer. 20 µl samples for each well were subjected to SDS-PAGE.

### Statistical Analysis

All of the values are expressed as mean ± SD. Differences between 2 groups were determined by a Student *t* test. Comparisons between groups on Western blotting data were assessed by 1-way ANOVA followed by a Bonferroni correction. A value of *p*<0.05 was considered statistically significant.

## Results

### Up-regulation of UCH-L1 in pressure overloaded hearts

**Figure 1:**
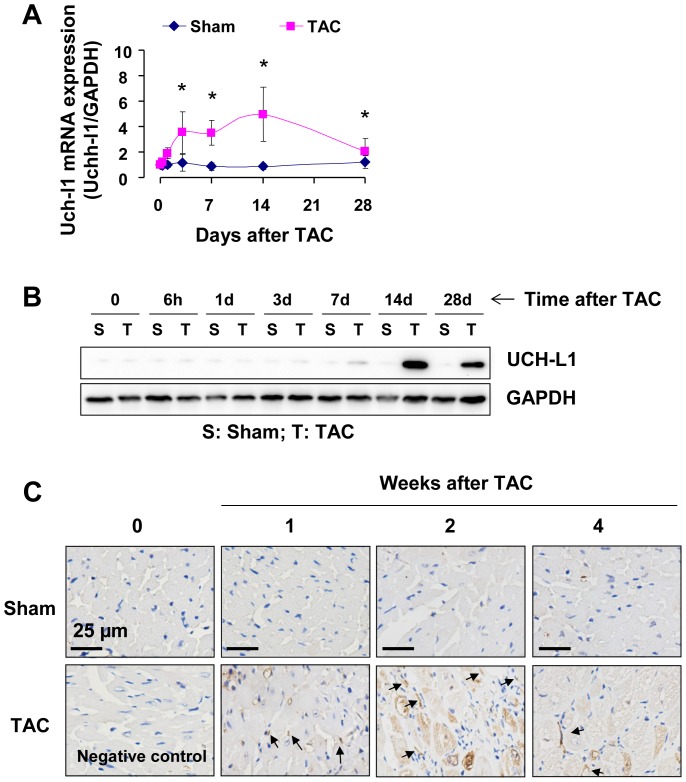
Expression of UCH-L1 in the heart after TAC. **A**. UCH-L1 mRNA expression in the left ventricles of mice after sham and TAC. n = 6, *p<0.05 vs. sham controls. **B**. Western blot analysis of UCH-L1 expression in the left ventricles of mice after sham and TAC. n = 3. **C**. UCH-L1 staining in the left ventricles of mice after sham and TAC. The results are representatives of 4 separated experiments.

To study the potential role of UCH-L1 in the pathogenesis of maladaptive cardiac remodeling and heart failure, we initially examined UCH-L1 expression profile in murine hearts after TAC, a well-established model of pressure overload-induced cardiac remodeling and dysfunction in rodents as previously described [12]. In the wild-type male C57BL/6J mice, TAC gradually induced cardiac hypertrophy that was characterized by an increase in the HW/BW ratio and cardiomyocyte size, induction of ANF, BNP and β-MHC fetal genes, and thickening of the diastolic left ventricle posterior wall (LVPW;d) (Figure S2A and C). Cardiac hypertrophy was evident by day 7 and progressive up to the experimental end point of 28 days (Figure S2A). In addition, a profound cardiac fibrosis was observed in the heart after TAC (Figure S2B). Fractional shortening FS (%) was preserved until day 14 and significantly decreased on day 28 (Figure S2A). Thus, we established a tissue bank of LV tissues at different time points after sham and TAC mice including the stages of adaptive cardiac remodeling with preserved cardiac function (days 1–14) and maladaptive cardiac remodeling with left ventricular dysfunction (days 14–28) [12]. Using the tissue bank, we observed that UCH-L1 mRNA expression was rapidly increased initially on day 1, reached a peak on day 14, and thereafter decreased to near to the basal level on day 28 after TAC (Figure 1A), while protein expression exhibited a similar pattern subsequently to the increases in UCH-L1 mRNA expression (Figure 1B and C). These results indicate that UCH-L1 expression are enhanced in the heart during the earlier stage of cardiac adaptive hypertrophy and declined in the process of maladaptive responses to the sustained hemodynamic stress, suggesting that UCH-L1 might play a critical role in the regulation of maladaptive cardiac remodeling and the transition of cardiac hypertrophy to heart failure.

### A growth inhibitory role of UCH-L1 in cardiac fibroblasts

Since UCH-L1 was upregulated in both cardiac myocytes and fibroblasts (Figure 1C), we questioned pathological significance of the UCH-L1 upregulation in maladaptive cardiac remodeling and dysfunction. With a focus, we determined a role of UCH-L1 in regulating proliferation of cardiac fibroblasts that play a critical role in cardiac fibrosis, a major contributor to cardiac dysfunction [14].

**Figure 2:**
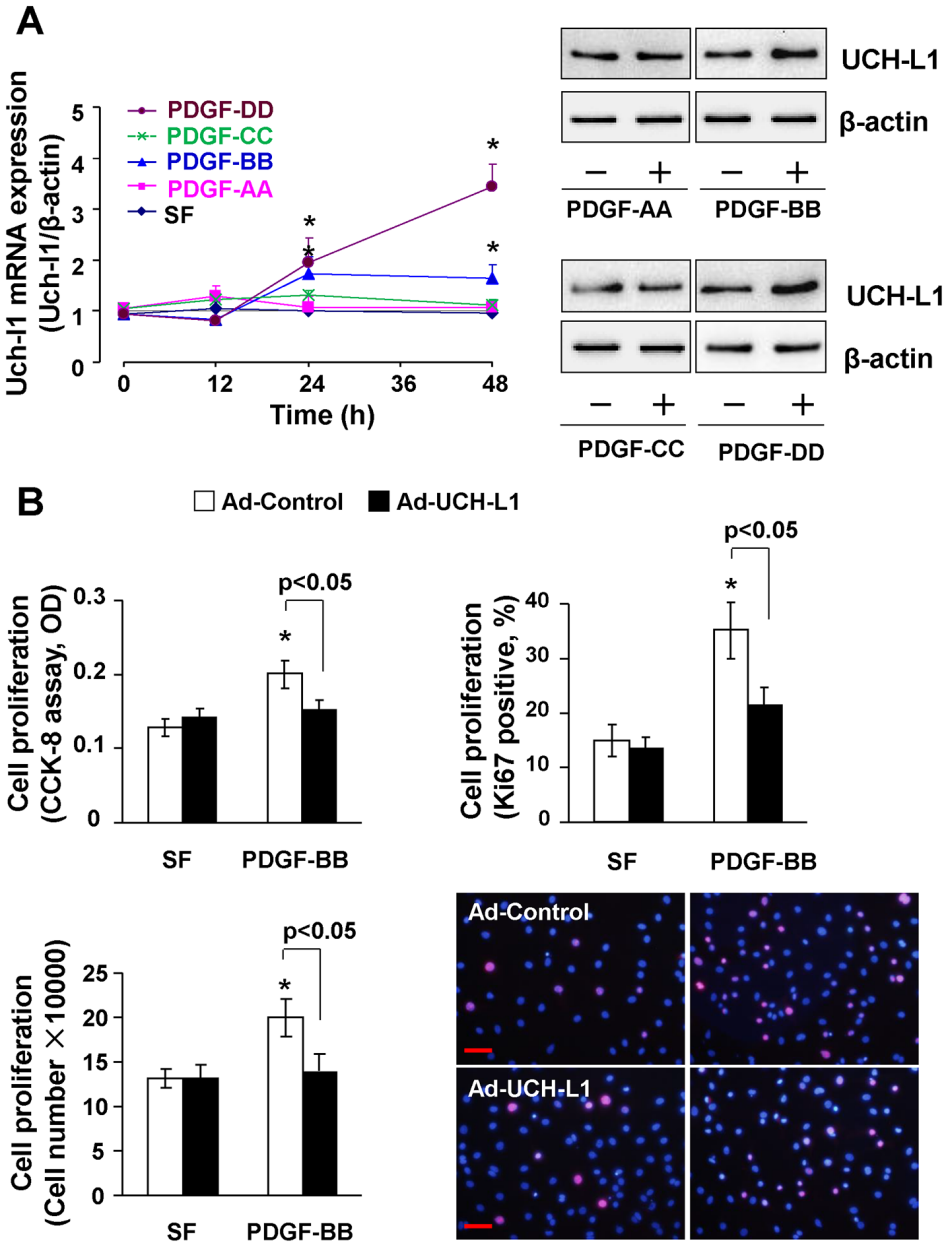
Role of UCH-L1 in regulating cardiac fibroblast proliferation. **A**. PDGF-induced UCH-L1 mRNA (*Left*) and protein (*Right*) expression in rat neonatal cardiac fibroblasts. Quiescent cells were treated with or without PDGF-AA (50 ng/ml), PGFD-BB (20 ng/ml), PDGF-CC (50 ng/ml), and PDGF-DD (50 ng/ml) for 24 h and subjected to Western blot analysis. n = 4, *p<0.05 vs SF controls. **B**. CCK-8, cell counting and Ki67 staining detected the role of UCH-L1 in regulating PDGF-induced rat neonatal cardiac fibroblast proliferation. n = 4, *p<0.05 vs. Ad-controls. Scale bar, 50 µm.

It has been firmly established that increases in the levels of circulating and/or local PDGFs are likely to be key driving forces for the abnormal proliferation of cardiac fibroblast culminating in cardiac fibrosis [15]. PDGFs are a family of disulphide-bonded dimeric isoforms that are coded by 4 genes: the classical PDGF-A and PDGF-B as well as the subsequently identified PDGF-C and PDGF-D [16]. All PDGF isoforms exert their biological functions by binding to and activating 2 receptor tyrosine kinases, PDGF receptor (PDGFR)-α and PDGFR-β. In general, PDGF-AA and PDGF-CC bind to PDGFRα while PDGF-BB and PDGF-DD act via PDGFR-β. Cardiac-specific overexpression of PDGF-A develops severe cardiac fibrosis with early lethality, whereas overexpression of the other isoforms induces cardiac fibrosis in mouse heart, followed by hypertrophy or dilated cardiomyopathy [16], [17]. Thus, we examined the effect of PDGF isoforms on UCH-L1 expression in primary culture of rat neonatal cardiac fibroblasts. Of interest, not PDGF-AA or PDGF-CC but PDGF-BB and PDGF-DD stimulated both mRNA and protein expression of UCH-L1 (Figure 2A). Considering the fact of PDGF-BB and PDGF-DD act via the same receptor of PDGFR-β [16], these results indicate a unique role of UCH-L1 in the control of PDGF/PDGFR-β signaling axis. Accordingly, UCH-L1 gain-of-function approach was applied to determine the role of UCH-L1 upregulation in regulating PDGF-BB-induced cardiac fibroblast proliferation. As figure 2B shown, adenoviral overexpression of UCH-L1 moderate suppressed PDGF-BB-induced increases in ki67 positive cell numbers and total cell numbers of cardiac fibroblasts. Flow cytometry analysis revealed that the UCH-L1 overexpression inhibited PDGF-BB-induced increase in the transition from G1 to S phase in cardiac fibroblasts (Figure S3). Of note, it was PDGF-BB and PDGF-DD but not PDGF-AA or PDGF-CC that stimulated proliferation of rat neonatal cardiac fibroblasts (Figure S4A). To further explore the pathological relevance of UCH-L1-mediated growth inhibitory effect, we determined the impact of UCH-L1 overexpression on the proliferation of adult cardiac fibroblasts isolated from rabbits, which are phylogenetically closer to primates than mice and rats [18]. We observed that overexpression of UCH-L1 exerted a similar growth inhibitory effect on PDGF-BB- and PDGF-DD-induced proliferation in these cells (Figure S4B). These results revealed a growth inhibitory effect of UCH-L1 in cardiac fibroblasts of various species, presumably including the human. Because UCH-L1 overexpression did not affect hydroperoxide (H_2_O_2_)-induced cell death in cardiac fibroblasts (Figure S5), the UCH-L1-mediated growth inhibitory effect may be independent of cell death. Moreover, in our previous study, we demonstrated that overexpression of UCH-L1 does not affect PDGF-BB-induced proliferation of vascular smooth muscle cell [19]. In contrast, recent evidence has also revealed that UCH-L1 enhances proliferation of multiple cell types, including Hela cells, Neuro2a cells, and human cancer cell lines H727 and MCF [20]. Therefore, these results suggest that the UCH-L1-mediated growth inhibitory is most likely cardiac fibroblast specific.

### An UCH-L1-mediated enhancement of PDGF-BB-induced p21^WAF1/Cip1^ expression in cardiac fibroblasts

**Figure 3:**
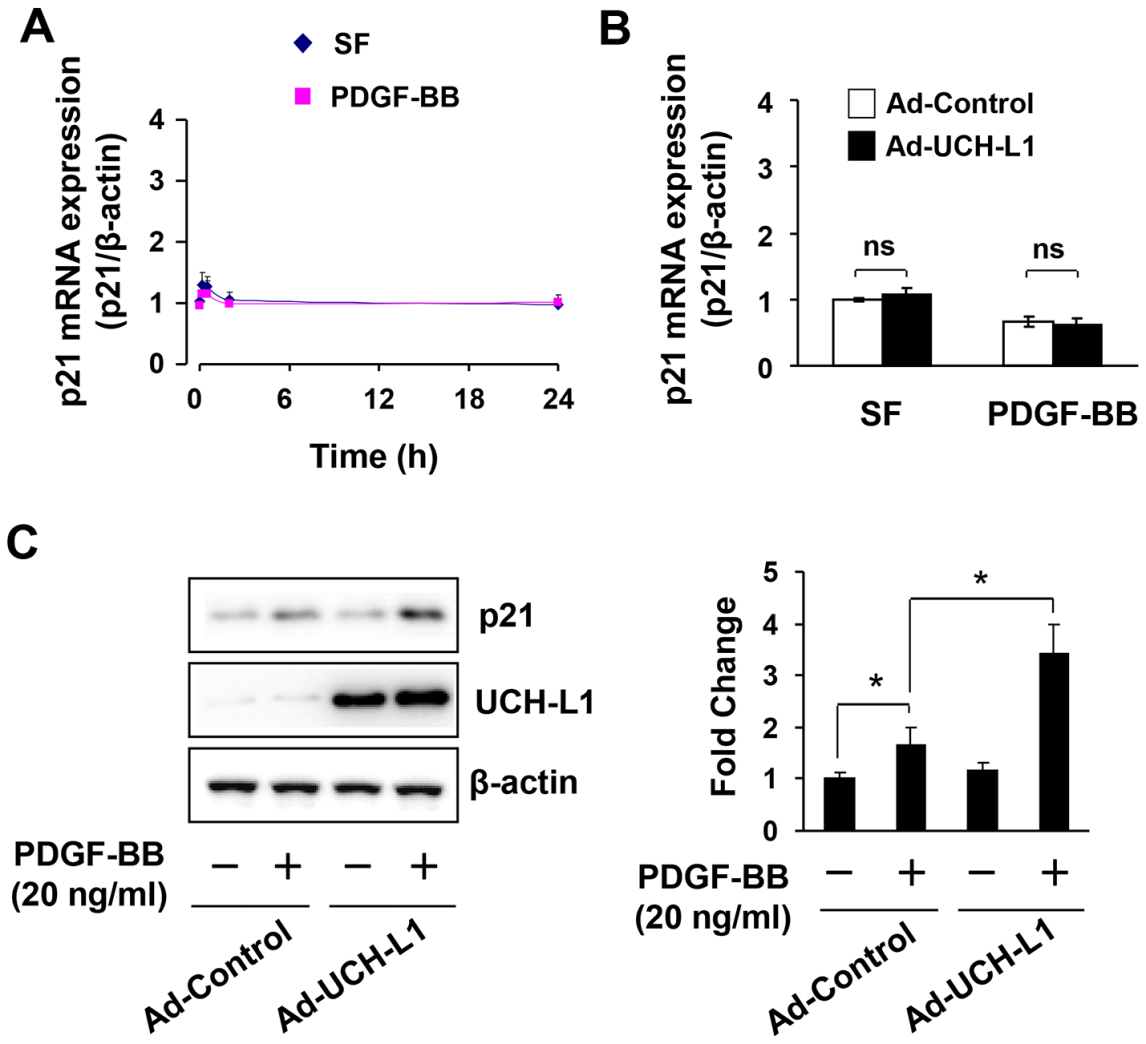
PDGF-induced p21^WAF1/Cip1^ (p21) expression. **A**. PDGF-induced p21 mRNA expression in rat neonatal cardiac fibroblasts. n = 4. **B**. Effect of adenoviral UCH-L1 overexpression on PDGF-induced p21 mRNA expression in rat neonatal cardiac fibroblasts. Quiescent cells were treated with or without PDGF-BB (20 ng/ml) for 6 h. n = 4. ns, no statistical significance. **C**. Effect of adenoviral UCH-L1 overexpression on PDGF-induced p21 protein expression in rat neonatal cardiac fibroblasts. Quiescent cells infected with Ad-control or Ad-UCH-L1 were treated with or without PDGF-BB (20 ng/ml) for 24 h. n = 4, *p<0.05. The results are representatives of 4 separated experiments.

To determine the molecular mechanism by which UCH-L1 suppresses cardiac fibroblast proliferation, we examined the effect of adenoviral overexpression of UCH-L1 on PDGF-BB-induced activation of MAPKs including extracellular signal-regulated kinase (ERK), c-Jun N-terminal kinases (JNK) and p38, phosphoinositide 3-kinase (PI3K), and signal transducers and activators of transcription 3 (STAT3), that are linked to cardiac fibroblast growth [16]. Surprisingly, none of the growth promoting signal cascades was affected by UCH-L1 overexpression (Figure S6). Thus, we further studied whether UCH-L1 regulates the downstream events of the mitogen signaling with a focus on cyclin-dependent kinases (CDKs), cyclin, and CDK inhbitors (CKIs), which either positively or negatively control the cell cycle progression [21]. Since the role of PDGF-BB in regulating functions of CDKs, cyclins, and CKIs in cardiac fibroblasts is unclear, we characterized the effect of PDGF-BB on the expression profile of CDKs and cyclins including CDK2, CDK4, cyclin D1, and cyclin E, as well as CKIs including p27^kip1^ and p21^WAF1/Cip1^ in primary culture of rat neonatal cardiac fibroblasts (Figure S6). We performed a detailed time-course study with harvesting cardiac fibroblasts at 0, 0.5, 2, 8, 24, and 48 hours after PDGF-BB stimulation. We observed that PDGF-BB regulated the expression of p21^WAF1/Cip1^ alone via a mechanism of posttranscriptional regulation (Figure 3, Figure S6). Of interest, overexpression of UCH-L1 enhanced the PDGF-BB-induced posttranscriptional upregulation of p21^WAF1/Cip1^ protein in cardiac fibroblasts (Figure 3), suggesting that UCH-L1 inhibits cardiac fibroblast proliferation via posttranscriptional enhancing the expression of p21^WAF1/Cip1^ to suppress the transition of G1 to S phase.

### UCH-L1 enhances PDGF-BB-induced upregulation of p21^WAF1/Cip1^ proteins via suppressing autophagic clearance of p21^WAF1/Cip1^ independent of ubiquitin-proteasomal system (UPS)-mediated protein degradation

**Figure 4:**
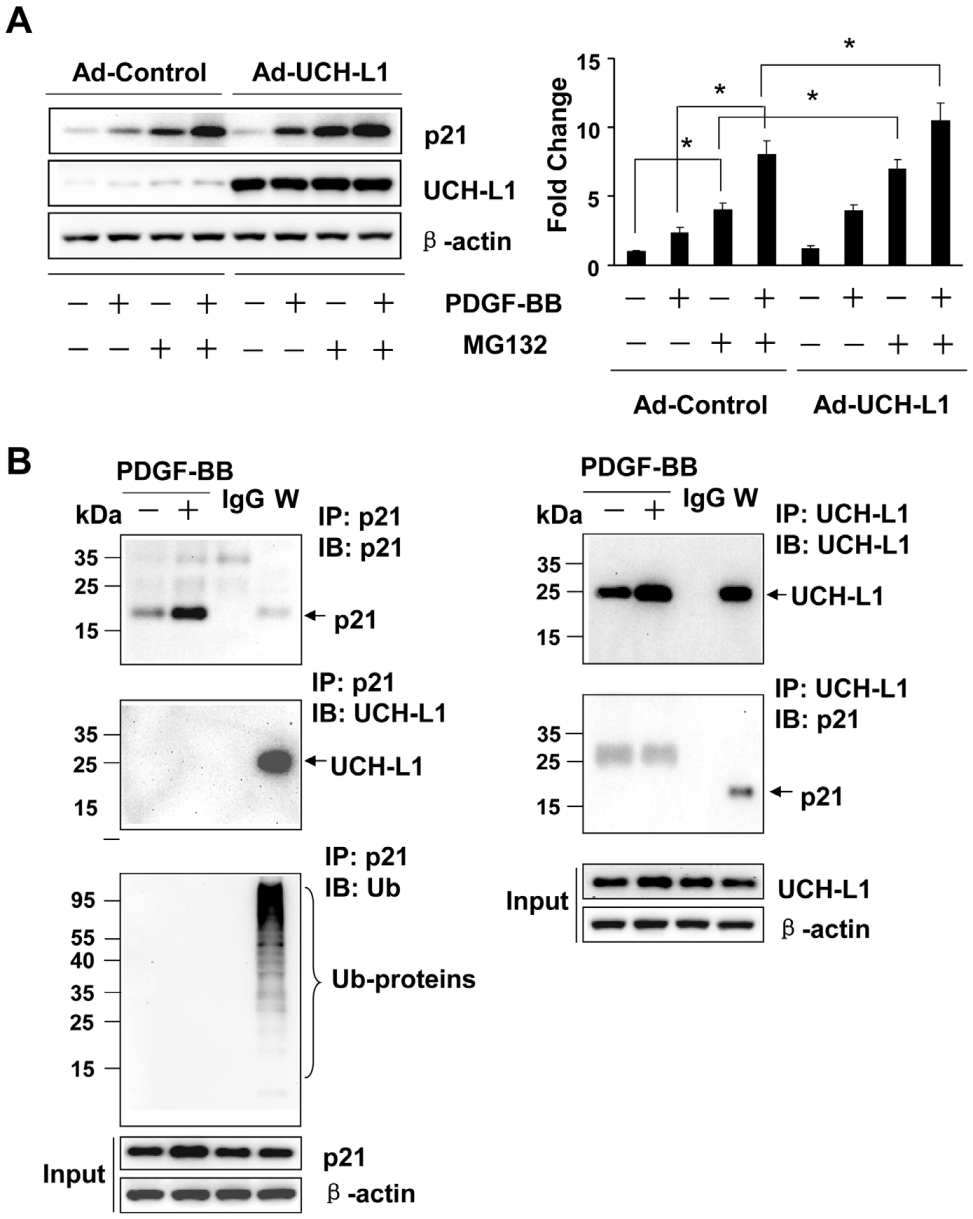
Effect of adenoviral UCH-L1 overexpression on ubiquitin proteasome system (UPS)-mediated degradation of p21^WAF1/Cip1^ (p21) proteins in cardiac fibroblasts. **A**. Effect of adenoviral UCH-L1 overexpression on MG132 and PDGF-induced upregulation of p21 protein expression in rat neonatal cardiac fibroblasts. Quiescent cells infected with Ad-control or Ad-UCH-L1 were treated with or without MG132 (0.5 µM) and PDGF-BB (20 ng/ml) for 24 h. Left panel: representatives of immunoblotting. Right panel: quantitatively densitometric analysis of protein expression. Data is presented as fold change of ratio of target protein to internal control β-actin relative to the Ad-Control (-). n = 4, *p<0.05. **B**. Effect of PDGF on p21 protein ubiquitination as well as interaction of UCH-L1 and p21 proteins in rat neonatal cardiac fibroblasts. Quiescent cells were treated with or without PDGF-BB (20 ng/ml) for 24 h. W, whole cell lysates; IP, immunoprecitated; IB, immunoblotted. Input, 10 µg of whole cell lysates subjected to IB. All results are representatives of at least 4 separated experiments.

**Figure 5:**
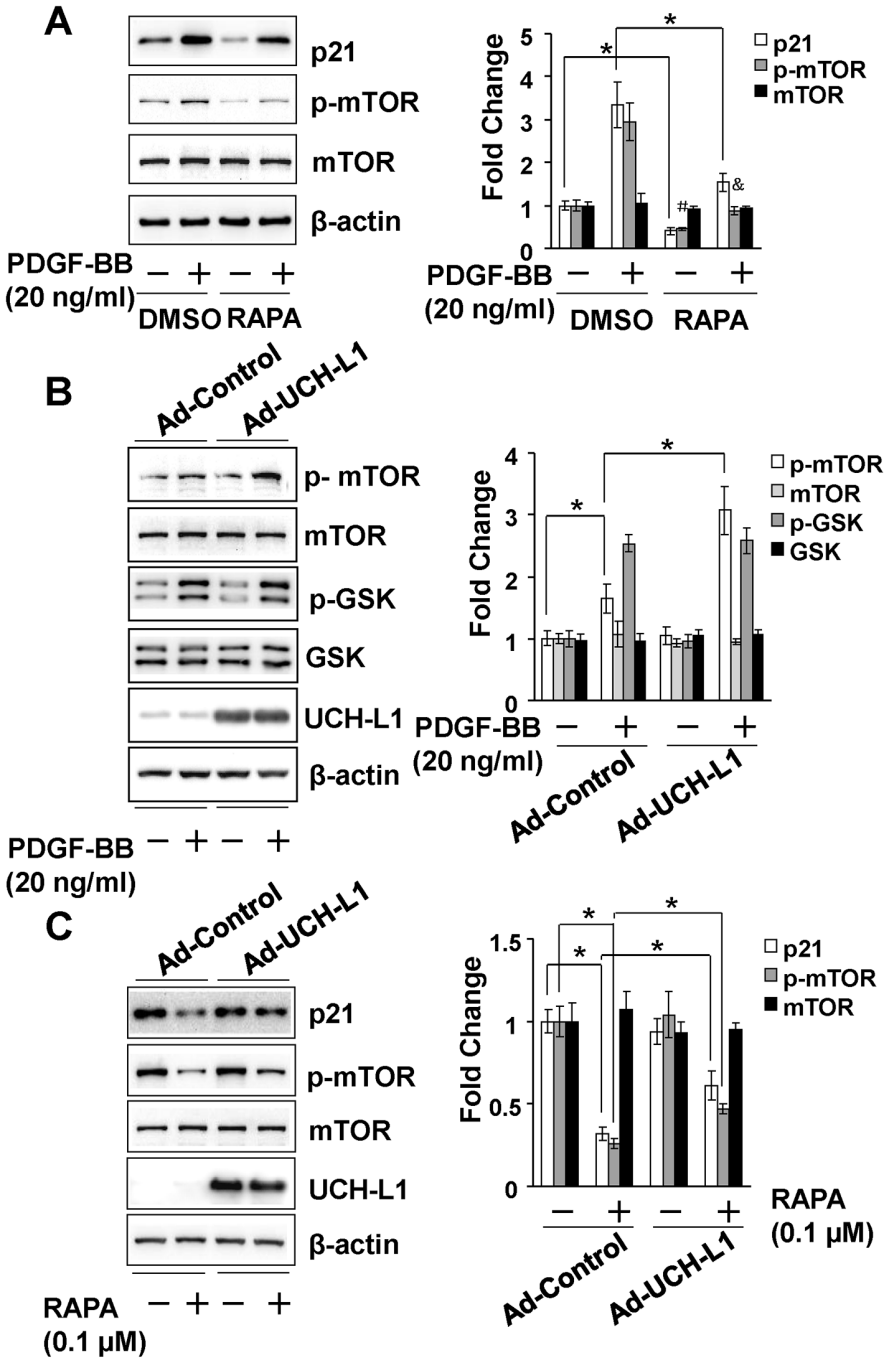
Role of autophagy in the control of p21^WAF1/Cip1^ (p21) protein expression in rat neonatal cardiac fibroblasts. Left panel: representatives of immunoblotting. Right panel: quantitatively densitometric analysis of protein expression. Data is presented as fold change of ratio of target protein to internal control β-actin relative to the Control (-). **A**. Effect of rapamycin on PDGF-BB-induced p21^WAF1/Cip1^ protein expression and mTOR phosphorylation. Quiescent cells were pretreated with rapamycin (RAPA, 0.1 µM) or vehicle DMSO for 1 h, and followed with co-treatment of RAPA (0.1 µM) and PDGF-BB (20 ng/ml) for additional 24 h. n = 4, *p<0.05. # p<0.05 vs. DMSO (-), & p<0.05 vs. DMSO (-) and PDGF-BB (-). **B**. Effect of UCH-L1 overexpression on PDGF-BB-induced phosphorylation of mTOR and GSK. Quiescent cells infected with Ad-control or Ad-UCH-L1 were treated with vehicle or PDGF-BB (20 ng/ml) for 2 h. n = 4, *p<0.05. **C**. Effect of UCH-L1 overexpression on rapamycin-induced suppression of mTOR phosphorylation and downregulation of p21^WAF1/Cip1^ protein expression. Quiescent cells infected with Ad-control or Ad-UCH-L1 were treated with vehicle or rapamycin (RAPA, 0.1 µM) for 24 h. n = 4, *p<0.05. All results are representatives of at least 4 separated experiments.

**Figure 6:**
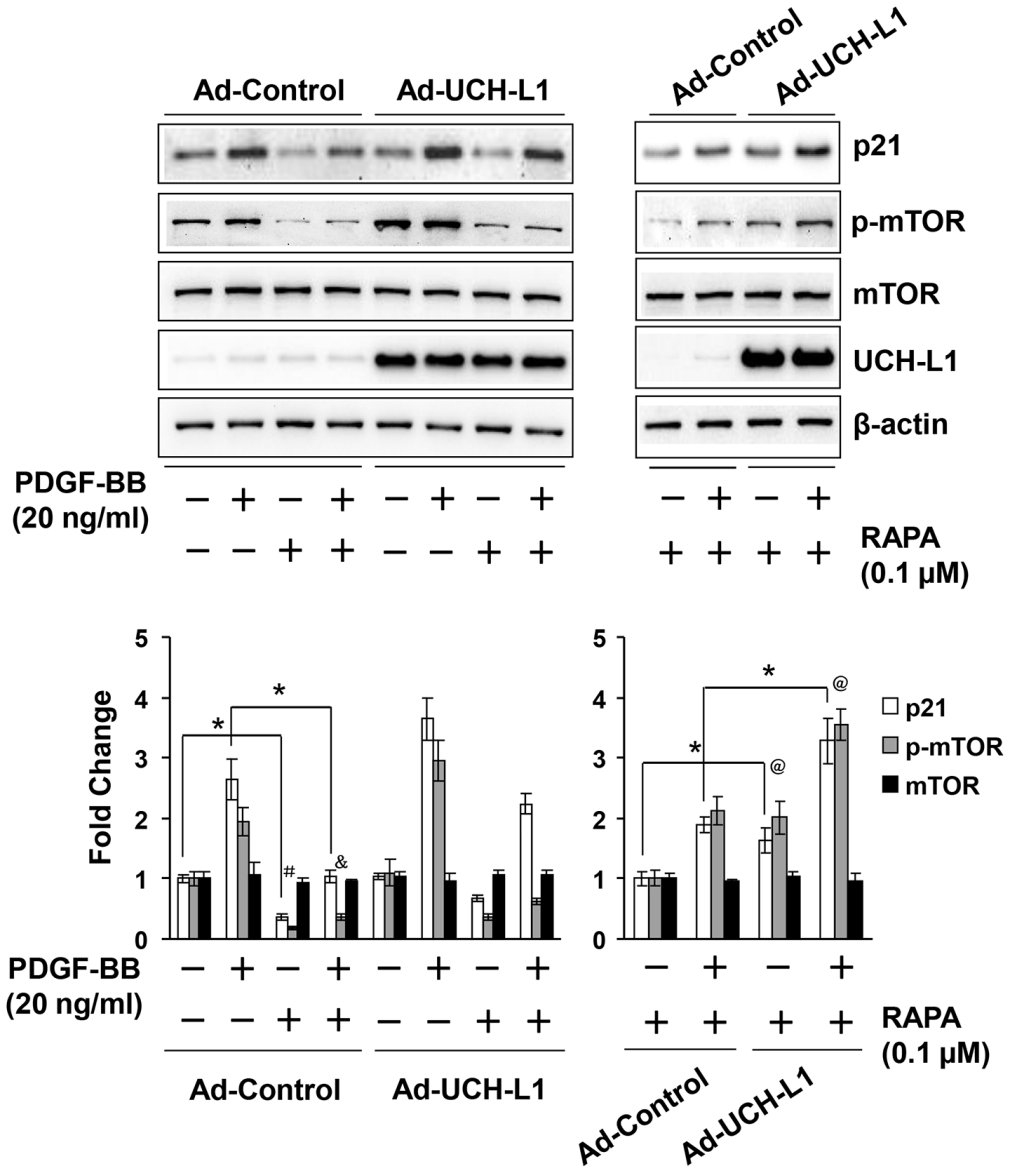
Role of UCH-L1 in the control of p21^WAF1/Cip1^ (p21) protein expression in the presence of rapamycin in rat neonatal cardiac fibroblasts. Effect of UCH-L1 overexpression on PDGF-BB-induced mTOR phosphorylation and upregulation of p21^WAF1/Cip1^ protein expression in the presence of rapamycin. Quiescent cells infected with Ad-control or Ad-UCH-L1 were pretreated with rapamycin (RAPA, 0.1 µM) or vehicle DMSO for 1 h, and followed with co-treatment of RAPA (0.1 µM) and PDGF-BB (20 ng/ml) for additional 24 h. Upper panel: representatives of immunoblotting. Lower panel: quantitatively densitometric analysis of protein expression. Data is presented as fold change of ratio of target protein to internal control β-actin relative to the Control (-) n = 4, *p<0.05. # p<0.05 vs. DMSO (-); & p<0.05 vs. DMSO (-) and PDGF-BB (+); @ p<0.05 vs. Ad-Controls. All results are representatives of at least 4 separated experiments.

**Figure 7:**
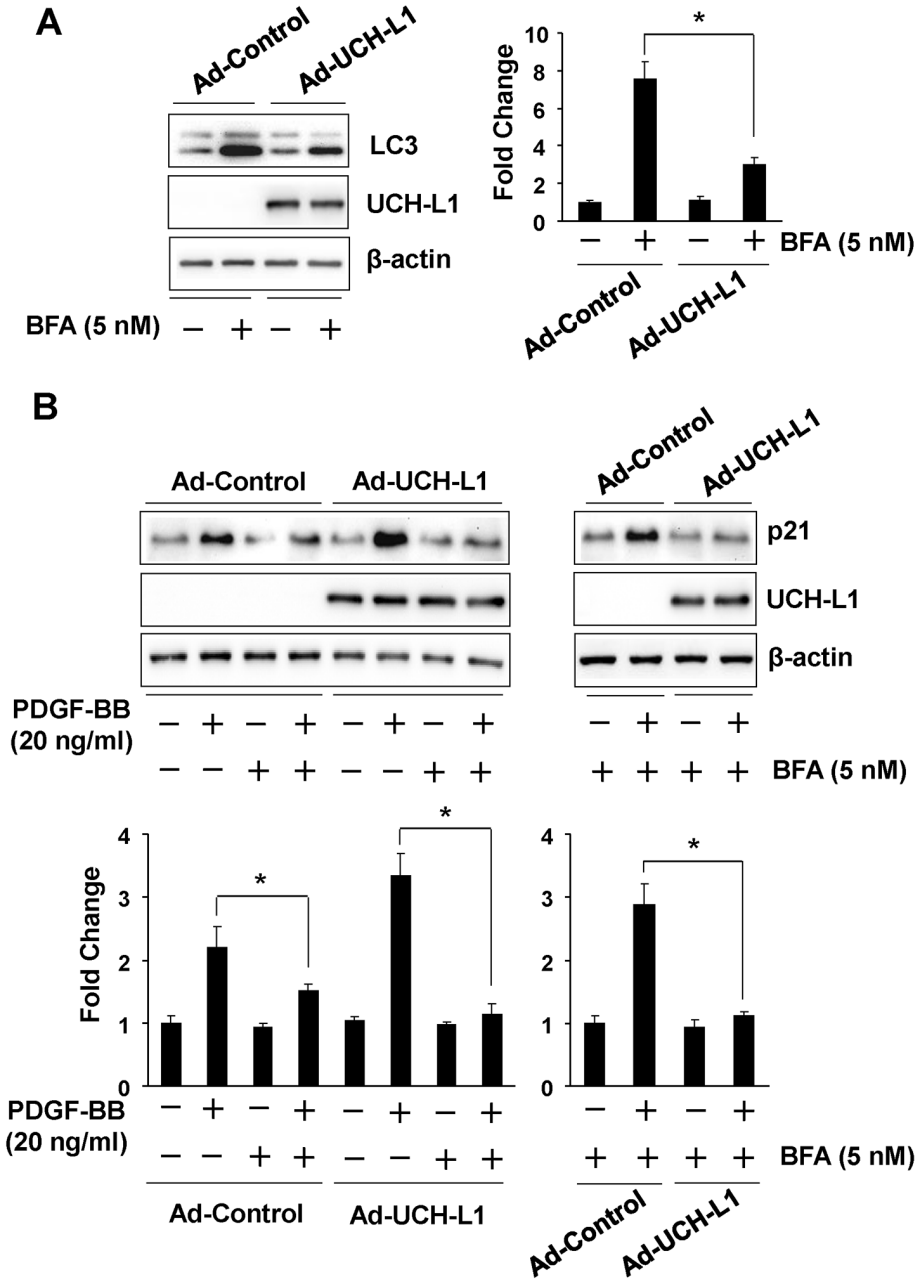
Role of UCH-L1 in autophagic clearance of p21^WAF1/Cip1^ in rat neonatal cardiac fibroblasts. **A**. Effect of adenoviral overexpression of UCH-L1 on bafilomycin A1 (BFA)-induced accumulation of LC3. Quiescent cells infected with adenovirus of GFP control or UCH-L1 were treated with or without BFA (5 nM) for 24 h. Left panel: representatives of immunoblotting. Right panel: quantitatively densitometric analysis of protein expression. Data is presented as fold change of ratio of target protein to internal control β-actin relative to the Control (-). n = 4, *p<0.05. **B**. Effect of BFA on UCH-L1-mediated upregulation of p21^WAF1/Cip1^. Quiescent infected cells as indicated were treated with or without PDGF-BB (20 ng/ml) in the presence of BFA (5 nM) for 24 h. Upper panel: representatives of immunoblotting. Lower panel: quantitatively densitometric analysis of protein expression. Data is presented as fold change of ratio of target protein to internal control β-actin relative to the Control (-). n = 4, *p<0.05. All results are representatives of at least 4 separated experiments.

To explore the underlying mechanism by which UCH-L1 enhances PDGF-BB-induced posttranscriptional upregulation of p21^WAF1/Cip1^, we determined a potential role of UCH-L1 in regulating p21 clearance by UPS and autophagy, two major pathways in the posttranscriptional control of protein levels [22]. Since MG132, a proteasome inhibitor, induced p21^WAF1/Cip1^ protein accumulation (Figure S7A), it is conceivable that UPS plays an important role in the regulation of p21^WAF1/Cip1^ protein level in cardiac fibroblasts. However, in the presence of MG132, PDGF-BB was still able to upregulate p21^WAF1/Cip1^ protein levels while overexpression of UCH-L1 enhanced not only the PDGF-BB-induced upregulation of p21^WAF1/Cip1^ protein in presence of MG132 but also the basal increased p21^WAF1/Cip1^ protein level induced by MG132 per se (Figure 4A, Figure S7B). Moreover, we did not observe physical association between UCH-L1 and p21^WAF1/Cip1^ proteins in cardiac fibroblast with or without PDGF-BB treatment. Unexpectedly, however, we did not find apparent ubiquitination of p21^WAF1/Cip1^ proteins in cardiac fibroblasts treated with or without PDGF-BB or MG132 (Figure 4B and Figure S7C). These results suggest that PDGF-BB upregulates p21^WAF1/Cip1^ protein expression via a mechanism independent of UPS-mediated protein degradation, and UCH-L1 enhances the PDGF-BB-induced UPS-independent upregulation of p21^WAF1/Cip1^ protein, although it may have an inhibitory effect on UPS-mediated degradation of p21^WAF1/Cip1^ protein in cardiac fibroblasts. Then, we investigated a potential involvement of autophagic protein degradation/clearance in the UCH-L1-mediated upregulation of p21^WAF1/Cip1^ protein in primary culture of cardiac fibroblasts. Rapamycin, an activator of autophagy via suppressing activity/phosphorylation of mammalian target of rapamycin (mTOR) [23], suppressed both basal and PDGF-BB-induced mTOR phosphorylation and p21^WAF1/Cip1^ protein expression (Figure 5A and Figure S8), suggesting a reversing relationship of autophagy activation and p21^WAF1/Cip1^ protein expression levels in cardiac fibroblasts. In addition, overexpression of UCH-L1 enhanced PDGF-BB-induced phosphorylation of mTOR but not glycogen synthase kinase-3 beta (GSK-3β) and attenuated rapamycin-induced suppression of mTOR phosphorylation (Figure 5B and C), indicating that a specific effect of UCH-L1 on mTOR activation is linked to the observed upregulation of p21^WAF1/Cip1^ protein expression. Moreover, UCH-L1 overexpression could recover the rapamycin-suppressed PDGF-BB-induced p21^WAF1/Cip1^ protein expression and mTOR phosphorylation (Figure 5C and Figure 6). These results suggest that UCH-L1 enhance PDGF-BB-induced p21^WAF1/Cip1^ protein expression via suppressing autophagy activation by increasing mTOR activity. To further address the notion, we applied bafilomycin A1 (BFA), an inhibitor of autophagic protein clearance [23], and examine overexpression of UCH-L1 on autophagic flux, a reliable assay to measure the capacity of autophagy-mediated protein clearance [23], and PDGF-BB-induced p21^WAF1/Cip1^ protein expression under a condition of BFA-impaired autophagic protein clearance in primary culture of rat neonatal cardiac fibroblasts. As figure 7A shown, treatment of BFA with a sub-toxic dose of 5 nM for 24 h induced accumulation of LC3, a biomarker reflecting amount of autophagosomes [23], in the control cells; however, this accumulation was attenuated in the Ad-UCH-L1 infected cells. BFA inhibits autophagosome fusion with lysosome thereby suppressing autophagic protein clearance [23], therefore, the BFA-induced accumulation is usually considered as the capacity of autophagy-mediated protein degradation, named autophagic flux. Accordingly, these results reveal that UCH-L1 suppresses autophagic protein clearance in cardiac fibroblast. Finally, we observed that UCH-L1 could not enhanced PDGF-BB-induced upregulation of p21^WAF1/Cip1^ protein expression anymore in the presence of BFA (figure 7B). Taken together, these findings demonstrate that UCH-L1 potentiates PDGF-BB-induced upregulation of p21^WAF1/Cip1^ protein expression via suppressing autophagic protein clearance in cardiac fibroblasts.

### Upregulation of p21^WAF1/Cip1^ in pressure overloaded heart

To explore pathological significance of UCH-L1-potentiated PDGF-BB-induced upregulation of p21^WAF1/Cip1^ in the heart, we examined the protein expression of p21^WAF1/Cip1^ in TAC hearts. We observed that there was a clear increase in p21^WAF1/Cip1^ protein expression when UCH-L1 upregulation reached a peak (Figure 1 and Figure S9). These results suggest that UCH-L1 facilitates autophagic degradation of p21^WAF1/Cip1^ to suppress proliferation of cardiac fibroblasts in chronically pressure overloaded heart, potentially acting as a novel feedback mechanism in the regulation of maladaptive cardiac remodeling and dysfunction.

## Discussion

In the present study, there are several novel findings regarding UCH-L1 as a negative regulator of maladaptive cardiac remodeling and dysfunction as follows: (i) UCH-L1 expression is enhanced in cardiac myocytes and fibroblasts during the earlier stage of cardiac adaptive hypertrophy and declined in the process of maladaptive responses to the sustained hemodynamic stress; (ii) UCH-L1 inhibits cardiac fibroblast proliferation via suppressing PDGF/PDGFRβ signaling; (iii) UCH-L1 preferentially enhances PDGF-BB-induced suppression autophagic clearance of p21^WAF1/Cip1^ proteins in cardiac fibroblasts. (iiii) UCH-L1 plays a minimal role in regulating cardiac fibroblast death.

DUBs appear to be critical regulators of a multitude of biological processes such as proliferation, apoptosis, differentiation, and inflammation and involved in several human diseases including tumor, neurodegenerative and renal diseases [2], [24], [25]. Of note, A20 has not been directly linked to human cardiac diseases although its cardiac protective actions have been well documented in mice [24]. However, the observed upregulation of UCH-L1 is in the human heart with dilated cardiomyopathy. Thus, our results reveal for the first time a contributory role of DUBs such as UCH-L1 in human cardiac diseases.

UCH-L1 (also known as protein gene product 9.5; pGp9.5) was originally described as a neuron-specific gene [26]. However, subsequent studies have also noted UCH-L1 expression in a wide variety of neuroendcrine cells including anterior pituicytes, thyroid parafollicular cells, pancreatic islet cells, and adrenal medullary cells as well as in a large number of nonneuroectodemrmally derived normal tissues including smooth muscle, prostatic secretory epithelium, ejaculatory duct cells, epididymis, mammary epithelial cells, Merkel cells, and dermal fibroblasts [27]. Several lines of evidence have suggested that UCH-L1 is involved not only in the pathogenesis of neurodegenerative diseases [28] but also in the regulation of tumor growth and spermatogenesis [29], [30]. In addition, we have demonstrated that UCH-L1 is expressed in vasculature and may acts as a negative regulator of vascular inflammatory responses and lesion formation [31]. It worthy to note that UCH-L1 suppresses tumor necrosis factor alpha (TNFα)- rather than PDGF-BB-mediated activation of ERK and proliferation in vascular smooth muscle cells (VSMCs) [19]. While UCH-L1 has been shown to inhibit α_2_-adrenergic receptor (AR) agonist-mediated activation of ERK via a direct association with α_2A_-AR receptor implicating a role of UCH-L1 in neuro-protection [32], it has also been documented that UCH-L1 up-regulates oncogenic β-catenin/TCF and Akt signaling to induce tumor cell proliferation and migration contributing to tumor progression [33], [34]. These results clearly demonstrate that UCH-L1 is a multi-functional protein and exerts cell type and/or tissue specific actions. Therefore, it was not surprising to find that UCH-L1 overexpression does not affect PDGF-BB-induced activation of MAPKs, Akt and STAT3 in cardiac fibroblasts; whereas, enhances PDGF-BB-induced posttransciptional upregulation of p21^WAF1/Cip1^ protein expression contributing to growth arrest in cardiac fibroblasts.

However, the finding of UCH-L1-suppressed autophagic clearance of p21^WAF1/Cip1^ protein independent of UPS-mediated protein degradation in cardiac fibroblasts is intriguing. Although autophagy is generally responsible for a bulky removal of less or insoluble protein aggregates and defective organelles [23], emerging evidence has indicated that autophagy plays an importantly role in the regulation of cellular signaling by providing autophagosomal membrance as a signaling platform or clearing the signaling complex per se via lysosomes [35], [36]. In fact, a potential role of autophagy in the regulation of p21^WAF1/Cip1^ expression has been evidenced by the rapamycin-induced posttranscriptional downregulation of p21^WAF1/Cip1^ protein expression and the BFA-induced accumulation of p21^WAF1/Cip1^ proteins in cancer cells [37]-[39]. In this context, our results further demonstrate that UCH-L1 is a critical inhibitor of autophagy-mediated clearance of p21^WAF1/Cip1^ proteins, serving as a novel feedback mechanism in the control of cardiac fibroblast growth.

It should be noted that UCH-L1 appears to be a key regulator of chaperone-mediated autophagy, which is essential for clearing and preventing the accumulation of alpha-synuclein, a cause of Parkinson disease [40]. Moreover, UCH-L1 is able to destabilize mTOR complex 1 (mTOCRC1) while increase mTORC2 activity toward Akt [41]. However, none of these mechanisms is likely attributable to the UCH-L1-mediated upregulation of p21^WAF1/Cip1^ protein expression in cardiac fibroblasts because: (i) UCH-L1 suppresses autophagy activity; (ii) UCH-L1 hardly regulates Akt activity. Considering the inhibitory effect of UCH-L1 on the suppression of mTOR phosphorylation induced by rapamycin which predominantly inhibits mTORC1 activity [42], it is plausible that UCH-L1 may operate a unique signaling to facilitate mTORC1-mediated autophagic clearance of p21^WAF1/Cip1^ protein specific in cardiac fibroblast aforementioned.

The precise molecular mechanisms of the UCH-L1-mediated upregulation of p21^WAF1/Cip1^ in cardiac fibroblasts have not been fully dissected in the present study. However, several studies have revealed that ubiquitination of mTOR, tuberous sclerosis complex (TSC), a component of mTORC1, or DEPTOR, a regulator of mTORC1, is linked to the control of mTORC1 activity [43]-[45]. Thus, whether UCH-L1 regulates these molecular events to suppress autophagic clearance of p21^WAF1/Cip1^ proteins deserves further investigation in cardiac fibroblasts. In addition, a direct link between the inhibitory effect of UCH-L1 on cardiac fibroblast growth and maladaptive cardiac remodeling and dysfunction needs to be addressed in future.

## Supporting Information

Figure S1 **Efficacy of adenoviral overexpression of control GFP and UCH-L1 in rat neonatal cardiac fibroblasts.** **A**. Microscopic analysis of cells expressing UCH-L1. Cells were infected with Ad-GFP or Ad-UCH-L1 at dose of 50 MOI for 48 h. **B**. Dose-dependent expression of UCH-L1. Cells infected with Ad-GFP or Ad-UCH-L1 at different MOIs as indicated for 48 h and then subjected to Western blot analysis.
(TIF)

Figure S2 **Transverse aortic constriction (TAC)-induced cardiac remodeling and dysfunction in adult male C57BL/6J mice.** Male C57BL/6J mice at ages of 8 weeks were subjected to sham or TAC operations. **A**. Heart weight/tibia length ratio (HW/Tibia), lung weight/tibia length ratio (LW/Tibia), and cardioechographic measuring of thickening of the diastolic left ventricle posterior wall (LVPW;d) and fractional shorting (FS) at different times after TAC. n = 15, *p<0.05 vs. normal or sham. **B**. WGA and Masson staining of left ventricles 4 weeks after TAC. Cardiomyocyte hypertrophy was determined by measuring crosssectional areas of cardiomyocytes which membranes were staining with WGA. Cardiac fibrosis was determined by measuring accumulated collagen fibers which were labeled by Masson. Results are representatives of 4 separated experiments (n = 8). **C**. qPCR analysis of fetal gene expression in the heart 4 weeks after TAC. n = 5, *p<0.05 vs. normal or sham.
(TIF)

Figure S3 **Flow cytometry analysis of cell cycle progression in rat neonatal cardiac fibroblasts.** Cells were infected with adenovirus of UCH-L1 or Control (MOI = 50) in serum free DMEM for 48 h, and then were stimulated with PDGF-BB (20 ng/ml) for additional 24 h. Cells were trypsinized, fixed and stained with Propidium (50 µg/ml), then detected by flow cytometry. n = 4. *p<0.05 vs. Ad-control. Results are representative of three independent experiments.
(TIF)

Figure S4 **Role of UCH-L1 in regulating PDGF-induced rat neonatal (A) and rabbit (B) cardiac fibroblast proliferation.** Quiescent cells infected with Ad-control or Ad-UCH-L1 were treated with or without PDGF-AA (50 ng/ml), PGFD-BB (20 ng/ml), PDGF-CC (50 ng/ml), and PDGF-DD (50 ng/ml) for 48 h and subjected to CCK-8 analysis. n = 4, *p<0.05 vs. Ad-controls.
(TIF)

Figure S5 **Effect of adenoviral overexpression of UCH-L1 on H_2_O_2_-induced cell death in rat neonatal cardiac fibroblasts.** Rat neonatal cardiac fibroblasts (passage 2) were seeded in 96-well plate with 10000 cells/well. Cells were infected with adenovirus of UCH-L1 or GFP (MOI = 50) in serum free DMEM for 48 h, and then were treated with H_2_O_2_ (H3410, Sigma) as indicated for 24 h. Cell death was assessed by LDH kit (Cat. No. 11644793001, Roche). Result was representative of four independent experiments.
(TIF)

Figure S6 **A. Effect of adenoviral overexpression of UCH-L1 on PDGF-BB-induced activation of MAPKs, Akt, and STAT3 in rat neonatal cardiac fibroblasts.** Rat neonatal cardiac fibroblasts (passage 2) at 90% confluent status were infected with adenovirus of UCH-L1 or GFP (MOI = 50) in serum free DMEM for 48 h, and then were stimulated with PDGF-BB (20 ng/ml) for 10 min. The cell lysates were subjected to Western blot analysis. Results are representatives of 4 separated experiments. **B. Effect of PDGF-BB on the expression of cell cycle regulators in rat neonatal cardiac fibroblasts.** Rat neonatal cardiac fibroblasts (passage 2) at 90% confluent status were cultured with serum free DMEM for 24 h to induce a quiescent status, and then stimulated with PDGF-BB (20 ng/ml) for different time periods as indicated. The cell lysates were subjected to Western blot analysis. Results are representatives of 4 separated experiments.
(TIF)

Figure S7 **Effect of adenoviral UCH-L1 overexpression on ubiquitin proteasome system (UPS)-mediated degradation of p21 proteins in cardiac fibroblasts.** **A**. Effect of MG132 on p21 protein expression in rat neonatal cardiac fibroblasts. Quiescent cells were treated with or without MG132 (0.5 µM) for 24 h. **B**. Effect of adenoviral UCH-L1 overexpression on PDGF-induced upregulation of p21 in the presence of MG132. Quiescent cells infected with Ad-control or Ad-UCH-L1 were treated with or without PDGF-BB (20 ng/ml) in the presence of MG132 (0.5 µM) for 24 h. Upper panel: representatives of immunoblotting. Lower panel: quantitatively densitometric analysis of protein expression. Data is presented as fold change of ratio of target protein to internal control β-actin relative to the Ad-Control PDGF-BB (-). n = 4, *p<0.05. **C**. Effect of MG132 on p21 protein ubiquitination as well as interaction of UCH-L1 and p21 proteins in rat neonatal cardiac fibroblasts. Quiescent cells were treated with or without MG132 (0.5 µM) for 24 h. W, whole cell lysates; IP, immunoprecitated; IB, immunoblotted. Input, 10 µg of whole cell lysates subjected to IB. All results are representatives of at least 4 separated experiments.
(TIF)

Figure S8 **Effect of PDGF-BB on mTOR activity.** Rat neonatal cardiac fibroblasts (passage 2) at 90% confluent status were cultured with serum free DMEM for 24 h to induce a quiescent status, and then stimulated with PDGF-BB (20 ng/ml) for different time periods as indicated. The cell lysates were subjected to Western blot analysis. Results are representatives of 4 separated experiments.
(TIF)

Figure S9 **Western blot analysis of p21 expression in the left ventricles of mice 2 weeks after sham and TAC.** n = 3. The results are representatives of 4 separated experiments.
(TIF)

Methods S1 **Supporting methods.**
(DOC)

## References

[1] Glickman MH, Ciechanover A (2002) The ubiquitin-proteasome proteolytic pathway: destruction for the sake of construction. Physiol Rev 82: 373–428. 11917093 10.1152/physrev.00027.2001

[2] Nijman SM, Luna-Vargas MP, Velds A, Brummelkamp TR, Dirac AM, et al (2005) A genomic and functional inventory of deubiquitinating enzymes. Cell 123: 773–786. 16325574 10.1016/j.cell.2005.11.007

[3] Mukhopadhyay D, Riezman H (2007) Proteasome-independent functions of ubiquitin in endocytosis and signaling. Science 315: 201–205. 17218518 10.1126/science.1127085

[4] Schulman BA, Harper JW (2009) Ubiquitin-like protein activation by E1 enzymes: the apex for downstream signalling pathways. Nat Rev Mol Cell Biol 10: 319–331. 19352404 10.1038/nrm2673 PMC2712597

[5] Willis MS, Patterson C (2006) Into the heart: the emerging role of the ubiquitin-proteasome system. J Mol Cell Cardiol 41: 567–579. 16949602 10.1016/j.yjmcc.2006.07.015

[6] Wang X, Robbins J (2006) Heart failure and protein quality control. Circ Res 99: 1315–1328. 17158347 10.1161/01.RES.0000252342.61447.a2

[7] Herrmann J, Lerman LO, Lerman A (2007) Ubiquitin and ubiquitin-like proteins in protein regulation. Circ Res 100: 1276–1291. 17495234 10.1161/01.RES.0000264500.11888.f0

[8] Patterson C, Ike C, Willis PWt, Stouffer GA, Willis MS (2007) The bitter end: the ubiquitin-proteasome system and cardiac dysfunction. Circulation 115: 1456–1463. 17372187 10.1161/CIRCULATIONAHA.106.649863

[9] Mearini G, Schlossarek S, Willis MS, Carrier L (2008) The ubiquitin-proteasome system in cardiac dysfunction. Biochim Biophys Acta 1782: 749–763. 18634872 10.1016/j.bbadis.2008.06.009

[10] Cook SA, Novikov MS, Ahn Y, Matsui T, Rosenzweig A (2003) A20 is dynamically regulated in the heart and inhibits the hypertrophic response. Circulation 108: 664–667. 12900338 10.1161/01.CIR.0000086978.95976.41

[11] Li HL, Zhuo ML, Wang D, Wang AB, Cai H, et al (2007) Targeted cardiac overexpression of A20 improves left ventricular performance and reduces compensatory hypertrophy after myocardial infarction. Circulation 115: 1885–1894. 17389268 10.1161/CIRCULATIONAHA.106.656835

[12] Li J, Ichikawa T, Villacorta L, Janicki JS, Brower GL, et al (2009) Nrf2 protects against maladaptive cardiac responses to hemodynamic stress. Arterioscler Thromb Vasc Biol 29: 1843–1850. 19592468 10.1161/ATVBAHA.109.189480 PMC12952473

[13] Cui TX, Nakagami H, Nahmias C, Shiuchi T, Takeda-Matsubara Y, et al (2002) Angiotensin II subtype 2 receptor activation inhibits insulin-induced phosphoinositide 3-kinase and Akt and induces apoptosis in PC12W cells. Mol Endocrinol 16: 2113–2123. 12198247 10.1210/me.2001-0284

[14] Manabe I, Shindo T, Nagai R (2002) Gene expression in fibroblasts and fibrosis: involvement in cardiac hypertrophy. Circ Res 91: 1103–1113. 12480810 10.1161/01.res.0000046452.67724.b8

[15] Leask A (2010) Potential therapeutic targets for cardiac fibrosis: TGFbeta, angiotensin, endothelin, CCN2, and PDGF, partners in fibroblast activation. Circ Res 106: 1675–1680. 20538689 10.1161/CIRCRESAHA.110.217737

[16] Andrae J, Gallini R, Betsholtz C (2008) Role of platelet-derived growth factors in physiology and medicine. Genes Dev 22: 1276–1312. 18483217 10.1101/gad.1653708 PMC2732412

[17] Ponten A, Folestad EB, Pietras K, Eriksson U (2005) Platelet-derived growth factor D induces cardiac fibrosis and proliferation of vascular smooth muscle cells in heart-specific transgenic mice. Circ Res 97: 1036–1045. 16224065 10.1161/01.RES.0000190590.31545.d4

[18] Hiripi L, Negre D, Cosset FL, Kvell K, Czompoly T, et al (2010) Transgenic rabbit production with simian immunodeficiency virus-derived lentiviral vector. Transgenic Res 19: 799–808. 20069454 10.1007/s11248-009-9356-y

[19] Ichikawa T, Li J, Dong X, Potts JD, Tang DQ, et al (2010) Ubiquitin carboxyl terminal hydrolase L1 negatively regulates TNFalpha-mediated vascular smooth muscle cell proliferation via suppressing ERK activation. Biochem Biophys Res Commun 391: 852–856. 19945429 10.1016/j.bbrc.2009.11.151 PMC3920551

[20] Kabuta T, Mitsui T, Takahashi M, Fujiwara Y, Kabuta C, et al (2013) Ubiquitin C-terminal hydrolase L1 (UCH-L1) acts as a novel potentiator of cyclin-dependent kinases to enhance cell proliferation independently of its hydrolase activity. J Biol Chem 288: 12615–12626. 23543736 10.1074/jbc.M112.435701 PMC3642309

[21] Sherr CJ, Roberts JM (1999) CDK inhibitors: positive and negative regulators of G1-phase progression. Genes Dev 13: 1501–1512. 10385618 10.1101/gad.13.12.1501

[22] Rubinsztein DC (2006) The roles of intracellular protein-degradation pathways in neurodegeneration. Nature 443: 780–786. 17051204 10.1038/nature05291

[23] Mizushima N, Yoshimori T, Levine B (2010) Methods in mammalian autophagy research. Cell 140: 313–326. 20144757 10.1016/j.cell.2010.01.028 PMC2852113

[24] Singhal S, Taylor MC, Baker RT (2008) Deubiquitylating enzymes and disease. BMC Biochem 9 Suppl 1 S3. 19007433 10.1186/1471-2091-9-S1-S3 PMC2582804

[25] Reyes-Turcu FE, Ventii KH, Wilkinson KD (2009) Regulation and cellular roles of ubiquitin-specific deubiquitinating enzymes. Annu Rev Biochem 78: 363–397. 19489724 10.1146/annurev.biochem.78.082307.091526 PMC2734102

[26] Wilkinson KD, Lee KM, Deshpande S, Duerksen-Hughes P, Boss JM, et al (1989) The neuron-specific protein PGP 9.5 is a ubiquitin carboxyl-terminal hydrolase. Science 246: 670–673. 2530630 10.1126/science.2530630

[27] Campbell LK, Thomas JR, Lamps LW, Smoller BR, Folpe AL (2003) Protein gene product 9.5 (PGP 9.5) is not a specific marker of neural and nerve sheath tumors: an immunohistochemical study of 95 mesenchymal neoplasms. Mod Pathol 16: 963–969. 14559978 10.1097/01.MP.0000087088.88280.B0

[28] Setsuie R, Wada K (2007) The functions of UCH-L1 and its relation to neurodegenerative diseases. Neurochem Int 51: 105–111. 17586089 10.1016/j.neuint.2007.05.007

[29] Liu Y, Lashuel HA, Choi S, Xing X, Case A, et al (2003) Discovery of inhibitors that elucidate the role of UCH-L1 activity in the H1299 lung cancer cell line. Chem Biol 10: 837–846. 14522054 10.1016/j.chembiol.2003.08.010

[30] Kwon J (2007) The new function of two ubiquitin C-terminal hydrolase isozymes as reciprocal modulators of germ cell apoptosis. Exp Anim 56: 71–77. 17460351 10.1538/expanim.56.71

[31] Takami Y, Nakagami H, Morishita R, Katsuya T, Cui TX, et al (2007) Ubiquitin carboxyl-terminal hydrolase L1, a novel deubiquitinating enzyme in the vasculature, attenuates NF-kappaB activation. Arterioscler Thromb Vasc Biol 27: 2184–2190. 17690318 10.1161/ATVBAHA.107.142505

[32] Weber B, Schaper C, Wang Y, Scholz J, Bein B (2009) Interaction of the ubiquitin carboxyl terminal esterase L1 with alpha(2)-adrenergic receptors inhibits agonist-mediated p44/42 MAP kinase activation. Cell Signal 21: 1513–1521. 19477270 10.1016/j.cellsig.2009.05.011

[33] Bheda A, Yue W, Gullapalli A, Whitehurst C, Liu R, et al (2009) Positive reciprocal regulation of ubiquitin C-terminal hydrolase L1 and beta-catenin/TCF signaling. PLoS One 4: e5955. 19536331 10.1371/journal.pone.0005955 PMC2694282

[34] Bheda A, Shackelford J, Pagano JS (2009) Expression and functional studies of ubiquitin C-terminal hydrolase L1 regulated genes. PLoS One 4: e6764. 19707515 10.1371/journal.pone.0006764 PMC2729380

[35] Young MM, Takahashi Y, Khan O, Park S, Hori T, et al (2012) Autophagosomal membrane serves as platform for intracellular death-inducing signaling complex (iDISC)-mediated caspase-8 activation and apoptosis. J Biol Chem 287: 12455–12468. 22362782 10.1074/jbc.M111.309104 PMC3320995

[36] Shi CS, Shenderov K, Huang NN, Kabat J, Abu-Asab M, et al (2012) Activation of autophagy by inflammatory signals limits IL-1beta production by targeting ubiquitinated inflammasomes for destruction. Nat Immunol 13: 255–263. 22286270 10.1038/ni.2215 PMC4116819

[37] Biecker E, De Gottardi A, Neef M, Unternahrer M, Schneider V, et al (2005) Long-term treatment of bile duct-ligated rats with rapamycin (sirolimus) significantly attenuates liver fibrosis: analysis of the underlying mechanisms. J Pharmacol Exp Ther 313: 952–961. 15769867 10.1124/jpet.104.079616

[38] Yohn NL, Bingaman CN, DuMont AL, Yoo LI (2011) Phosphatidylinositol 3'-kinase, mTOR, and glycogen synthase kinase-3beta mediated regulation of p21 in human urothelial carcinoma cells. BMC Urol 11: 19. 21864408 10.1186/1471-2490-11-19 PMC3173386

[39] Wu YC, Wu WK, Li Y, Yu L, Li ZJ, et al (2009) Inhibition of macroautophagy by bafilomycin A1 lowers proliferation and induces apoptosis in colon cancer cells. Biochem Biophys Res Commun 382: 451–456. 19289106 10.1016/j.bbrc.2009.03.051

[40] Kabuta T, Furuta A, Aoki S, Furuta K, Wada K (2008) Aberrant interaction between Parkinson disease-associated mutant UCH-L1 and the lysosomal receptor for chaperone-mediated autophagy. J Biol Chem 283: 23731–23738. 18550537 10.1074/jbc.M801918200 PMC3259779

[41] Hussain S, Feldman AL, Das C, Ziesmer SC, Ansell SM, et al (2013) Ubiquitin hydrolase UCH-L1 destabilizes mTOR complex 1 by antagonizing DDB1-CUL4-mediated ubiquitination of raptor. Mol Cell Biol 33: 1188–1197. 23297343 10.1128/MCB.01389-12 PMC3592026

[42] Wullschleger S, Loewith R, Hall MN (2006) TOR signaling in growth and metabolism. Cell 124: 471–484. 16469695 10.1016/j.cell.2006.01.016

[43] Zhao Y, Xiong X, Sun Y (2011) DEPTOR, an mTOR inhibitor, is a physiological substrate of SCF(betaTrCP) E3 ubiquitin ligase and regulates survival and autophagy. Mol Cell 44: 304–316. 22017876 10.1016/j.molcel.2011.08.029 PMC3216641

[44] Linares JF, Duran A, Yajima T, Pasparakis M, Moscat J, et al (2013) K63 Polyubiquitination and Activation of mTOR by the p62-TRAF6 Complex in Nutrient-Activated Cells. Mol Cell 51: 283–296. 23911927 10.1016/j.molcel.2013.06.020 PMC3971544

[45] Han S, Kim S, Bahl S, Li L, Burande CF, et al (2012) The E3 ubiquitin ligase protein associated with Myc (Pam) regulates mammalian/mechanistic target of rapamycin complex 1 (mTORC1) signaling in vivo through N- and C-terminal domains. J Biol Chem 287: 30063–30072. 22798074 10.1074/jbc.M112.353987 PMC3436263

