# Supplementary figures and images for: Ubiquitin Carboxyl Terminal Hydrolyase L1 -Suppressed Autophagic Degradation of p21WAF1/Cip1 as a Novel Feedback Mechanism in the Control of Cardiac Fibroblast Proliferation
Source: PLoS One. 2014 Apr 14;9(4):e94658. doi: 10.1371/journal.pone.0094658 (PMC3986084; doi:10.1371/journal.pone.0094658)

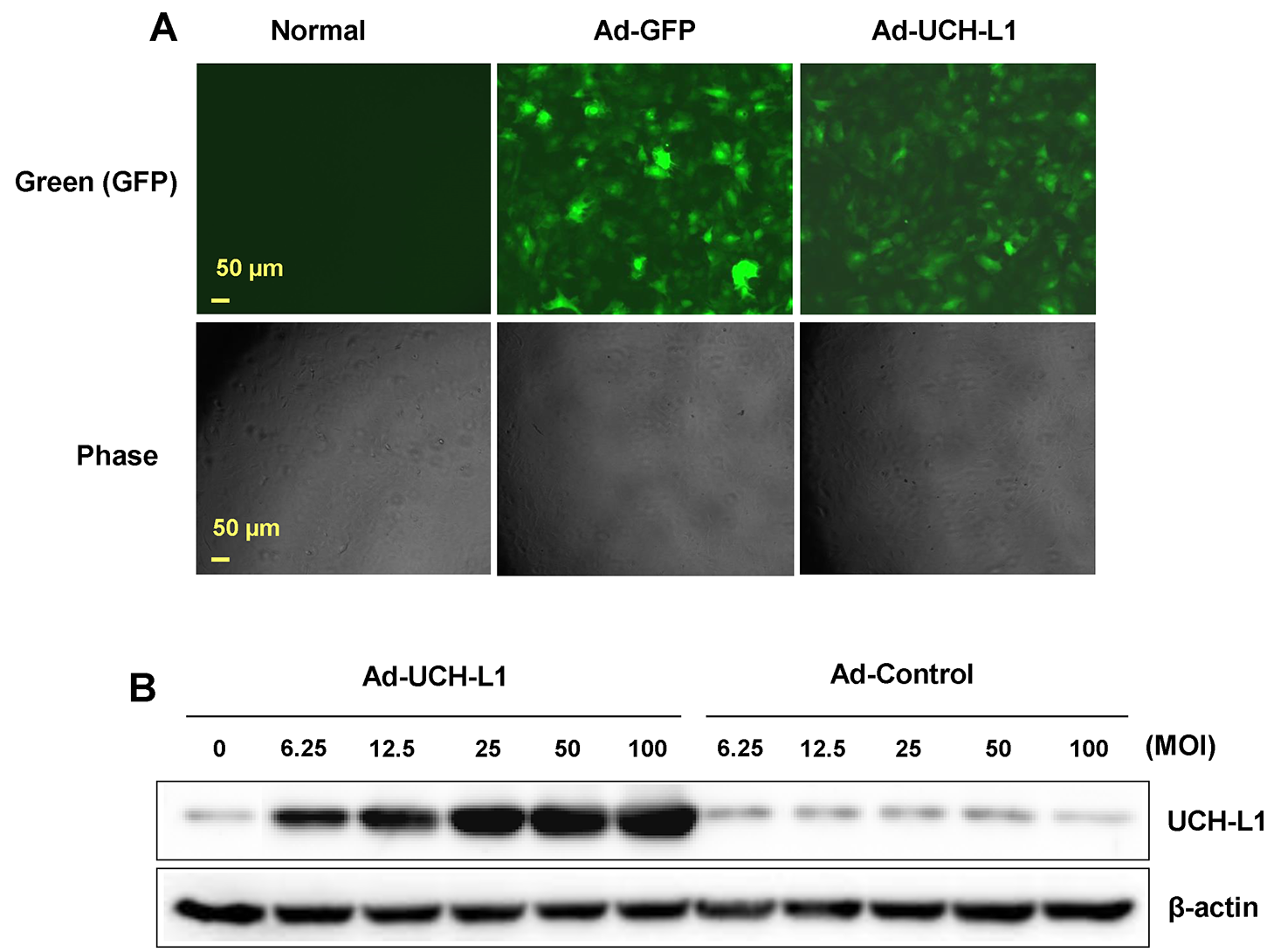

Supplement: Figure S1 — Efficacy of adenoviral overexpression of control GFP and UCH-L1 in rat neonatal cardiac fibroblasts. A. Microscopic analysis of cells expressing UCH-L1. Cells were infected with Ad-GFP or Ad-UCH-L1 at dose of 50 MOI for 48 h. B. Dose-dependent expression of UCH-L1. Cells infected with Ad-GFP or Ad-UCH-L1 at different MOIs as indicated for 48 h and then subjected to Western blot analysis. (TIF) [file pone.0094658.s001.tif]

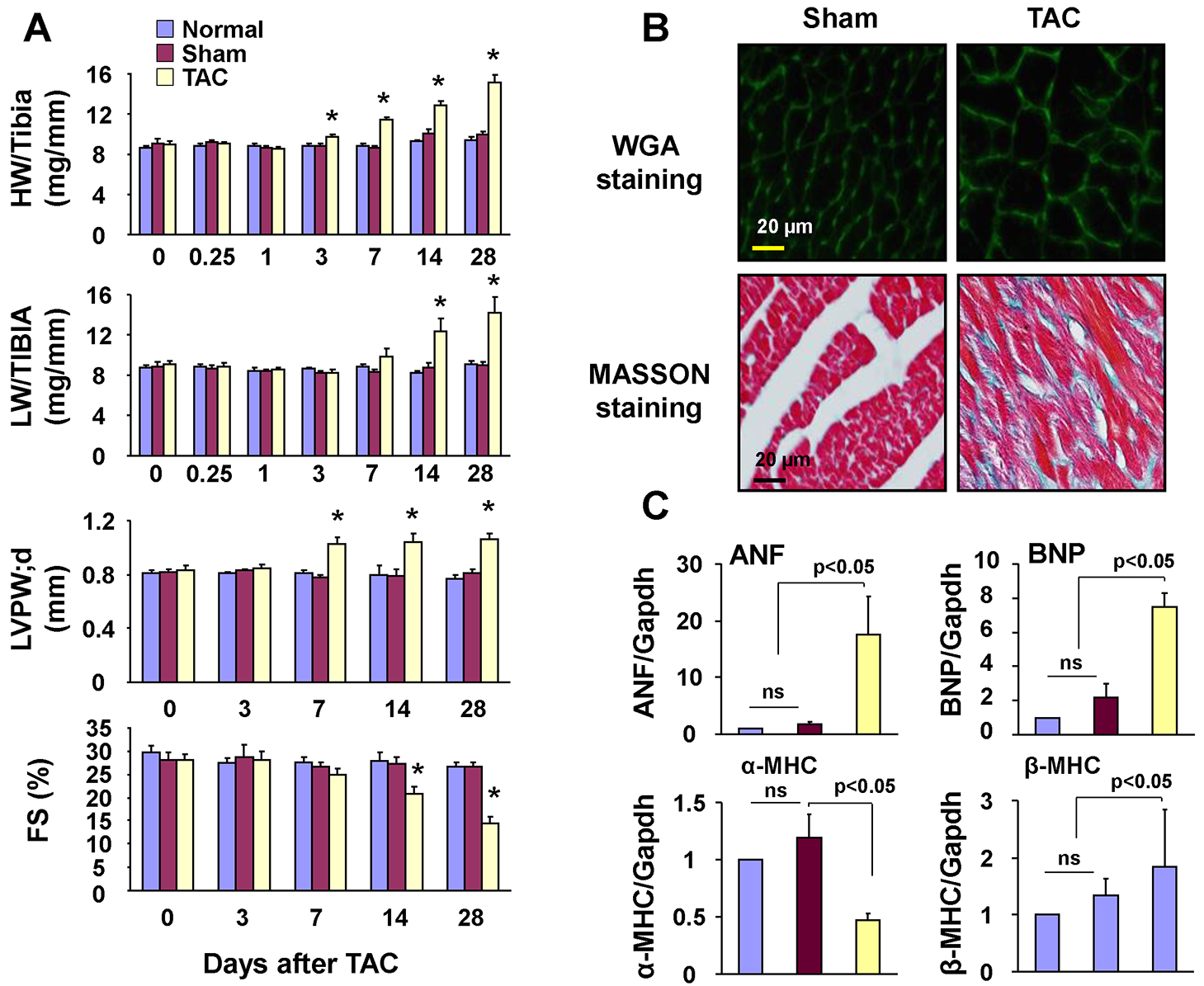

Supplement: Figure S2 — Transverse aortic constriction (TAC)-induced cardiac remodeling and dysfunction in adult male C57BL/6J mice. Male C57BL/6J mice at ages of 8 weeks were subjected to sham or TAC operations. A. Heart weight/tibia length ratio (HW/Tibia), lung weight/tibia length ratio (LW/Tibia), and cardioechographic measuring of thickening of the diastolic left ventricle posterior wall (LVPW;d) and fractional shorting (FS) at different times after TAC. n = 15, *p<0.05 vs. normal or sham. B. WGA and Masson staining of left ventricles 4 weeks after TAC. Cardiomyocyte hypertrophy was determined by measuring crosssectional areas of cardiomyocytes which membranes were staining with WGA. Cardiac fibrosis was determined by measuring accumulated collagen fibers which were labeled by Masson. Results are representatives of 4 separated experiments (n = 8). C. qPCR analysis of fetal gene expression in the heart 4 weeks after TAC. n = 5, *p<0.05 vs. normal or sham. (TIF) [file pone.0094658.s002.tif]

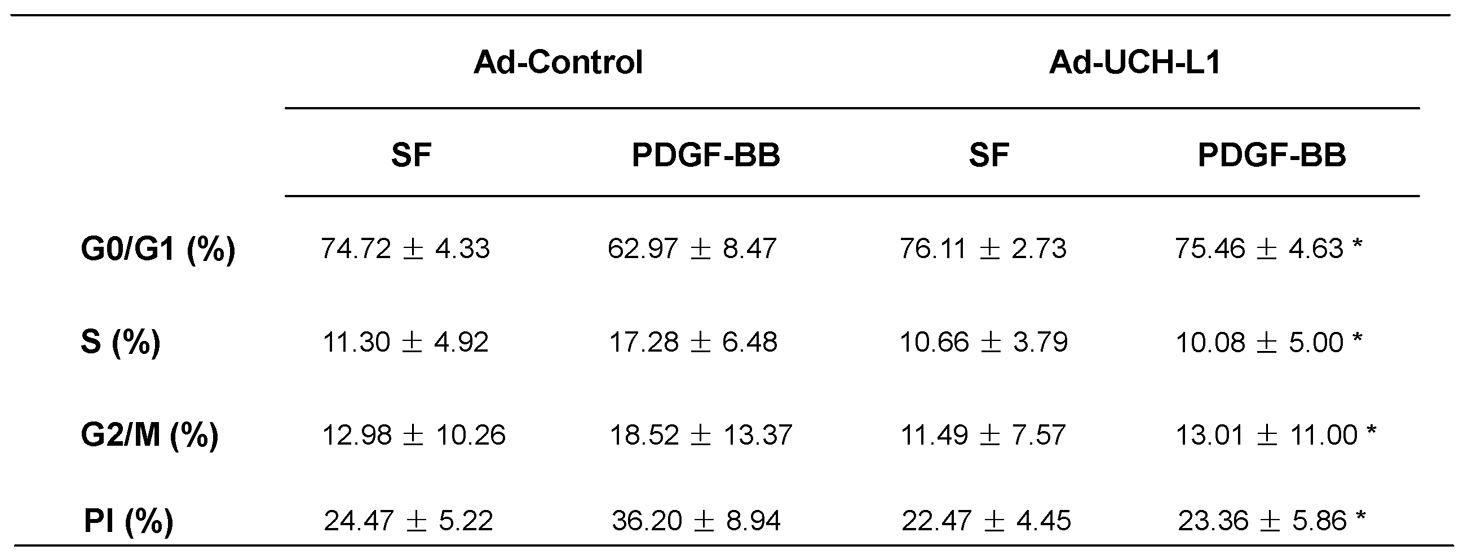

Supplement: Figure S3 — Flow cytometry analysis of cell cycle progression in rat neonatal cardiac fibroblasts. Cells were infected with adenovirus of UCH-L1 or Control (MOI = 50) in serum free DMEM for 48 h, and then were stimulated with PDGF-BB (20 ng/ml) for additional 24 h. Cells were trypsinized, fixed and stained with Propidium (50 µg/ml), then detected by flow cytometry. n = 4. *p<0.05 vs. Ad-control. Results are representative of three independent experiments. (TIF) [file pone.0094658.s003.tif]

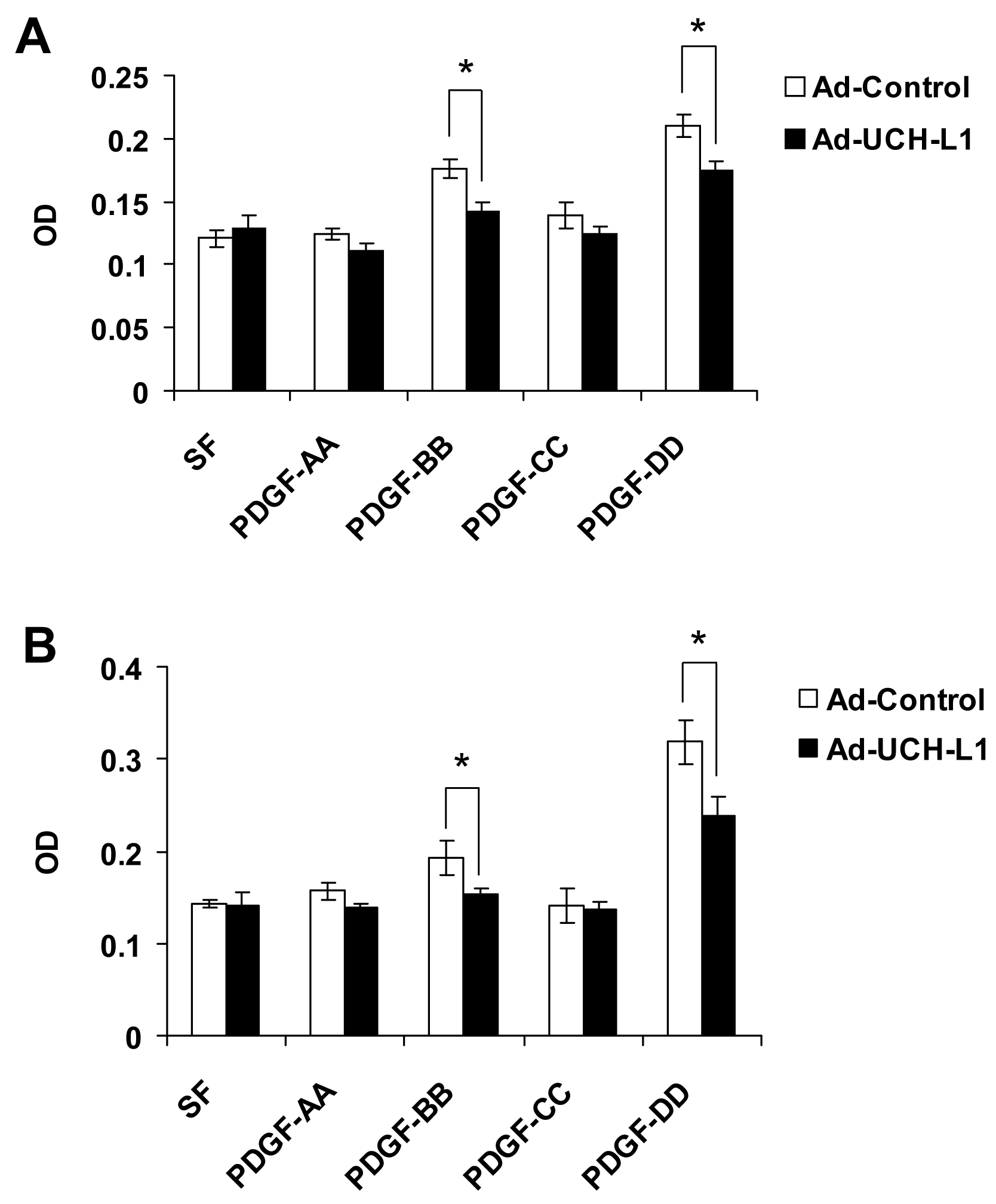

Supplement: Figure S4 — Role of UCH-L1 in regulating PDGF-induced rat neonatal (A) and rabbit (B) cardiac fibroblast proliferation. Quiescent cells infected with Ad-control or Ad-UCH-L1 were treated with or without PDGF-AA (50 ng/ml), PGFD-BB (20 ng/ml), PDGF-CC (50 ng/ml), and PDGF-DD (50 ng/ml) for 48 h and subjected to CCK-8 analysis. n = 4, *p<0.05 vs. Ad-controls. (TIF) [file pone.0094658.s004.tif]

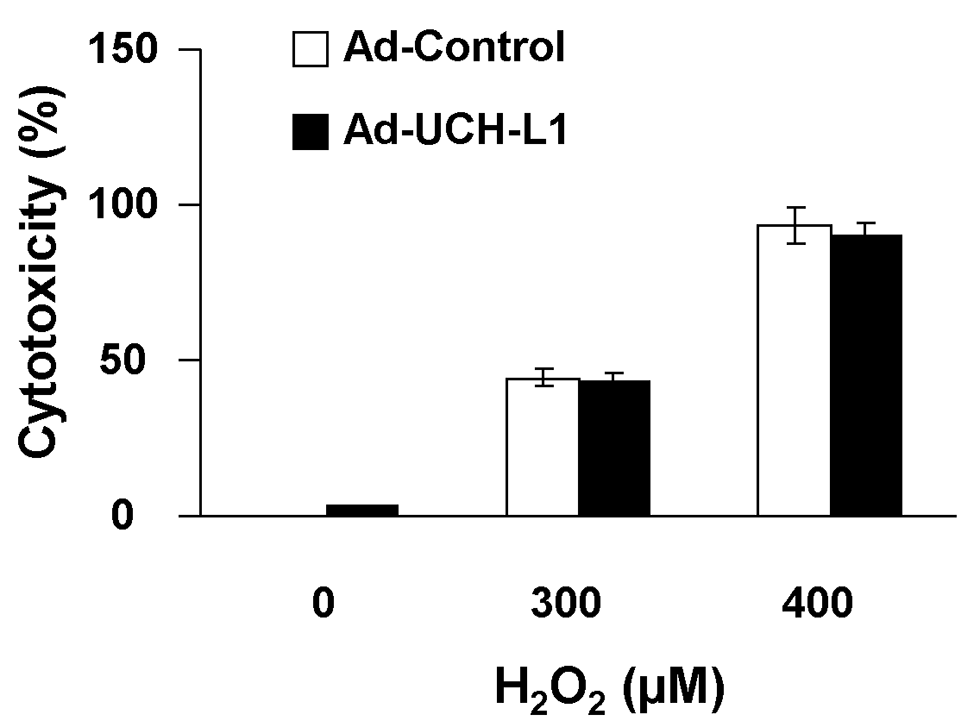

Supplement: Figure S5 — Effect of adenoviral overexpression of UCH-L1 on H2O2-induced cell death in rat neonatal cardiac fibroblasts. Rat neonatal cardiac fibroblasts (passage 2) were seeded in 96-well plate with 10000 cells/well. Cells were infected with adenovirus of UCH-L1 or GFP (MOI = 50) in serum free DMEM for 48 h, and then were treated with H2O2 (H3410, Sigma) as indicated for 24 h. Cell death was assessed by LDH kit (Cat. No. 11644793001, Roche). Result was representative of four independent experiments. (TIF) [file pone.0094658.s005.tif]

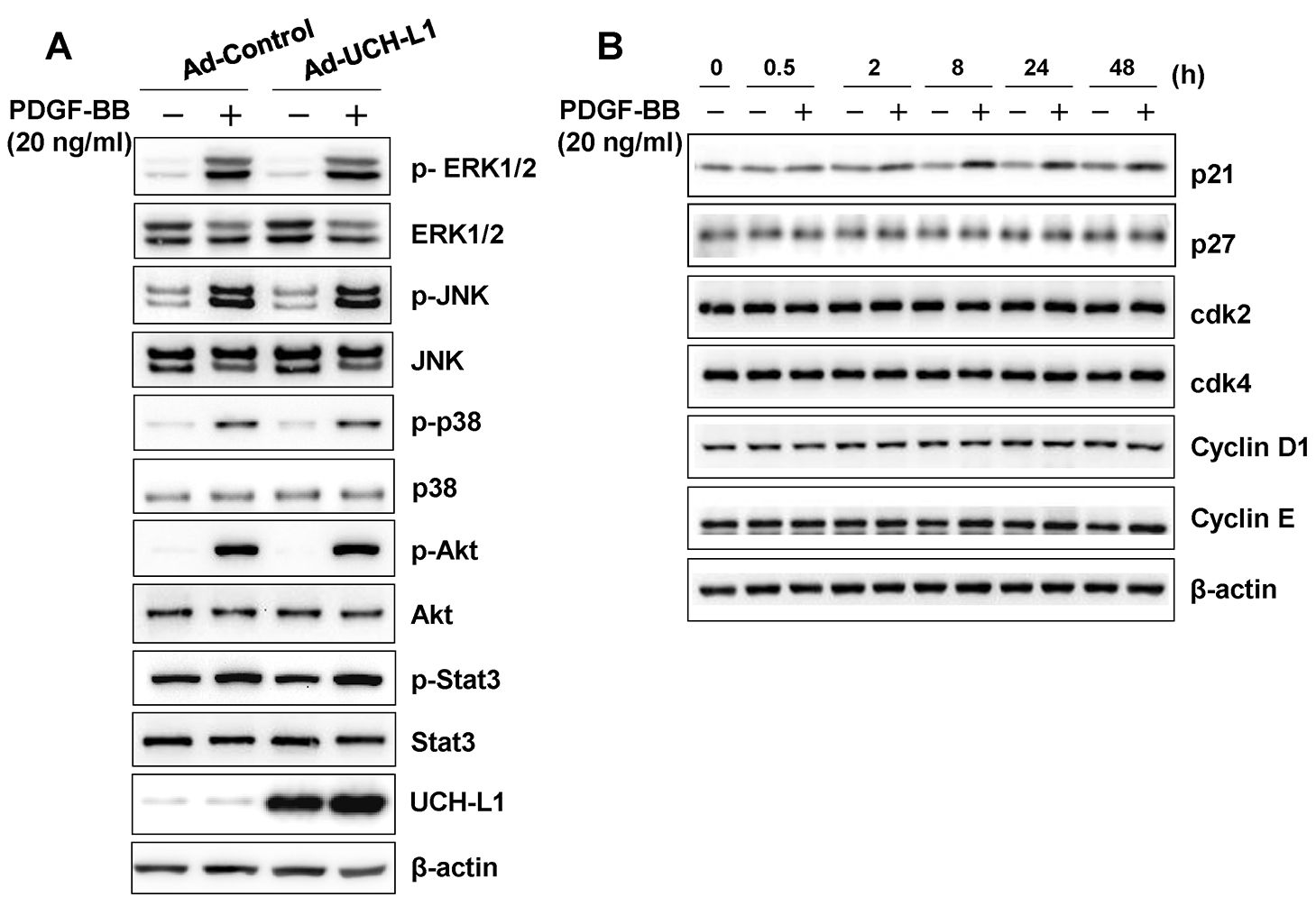

Supplement: Figure S6 — A. Effect of adenoviral overexpression of UCH-L1 on PDGF-BB-induced activation of MAPKs, Akt, and STAT3 in rat neonatal cardiac fibroblasts. Rat neonatal cardiac fibroblasts (passage 2) at 90% confluent status were infected with adenovirus of UCH-L1 or GFP (MOI = 50) in serum free DMEM for 48 h, and then were stimulated with PDGF-BB (20 ng/ml) for 10 min. The cell lysates were subjected to Western blot analysis. Results are representatives of 4 separated experiments. B. Effect of PDGF-BB on the expression of cell cycle regulators in rat neonatal cardiac fibroblasts. Rat neonatal cardiac fibroblasts (passage 2) at 90% confluent status were cultured with serum free DMEM for 24 h to induce a quiescent status, and then stimulated with PDGF-BB (20 ng/ml) for different time periods as indicated. The cell lysates were subjected to Western blot analysis. Results are representatives of 4 separated experiments. (TIF) [file pone.0094658.s006.tif]

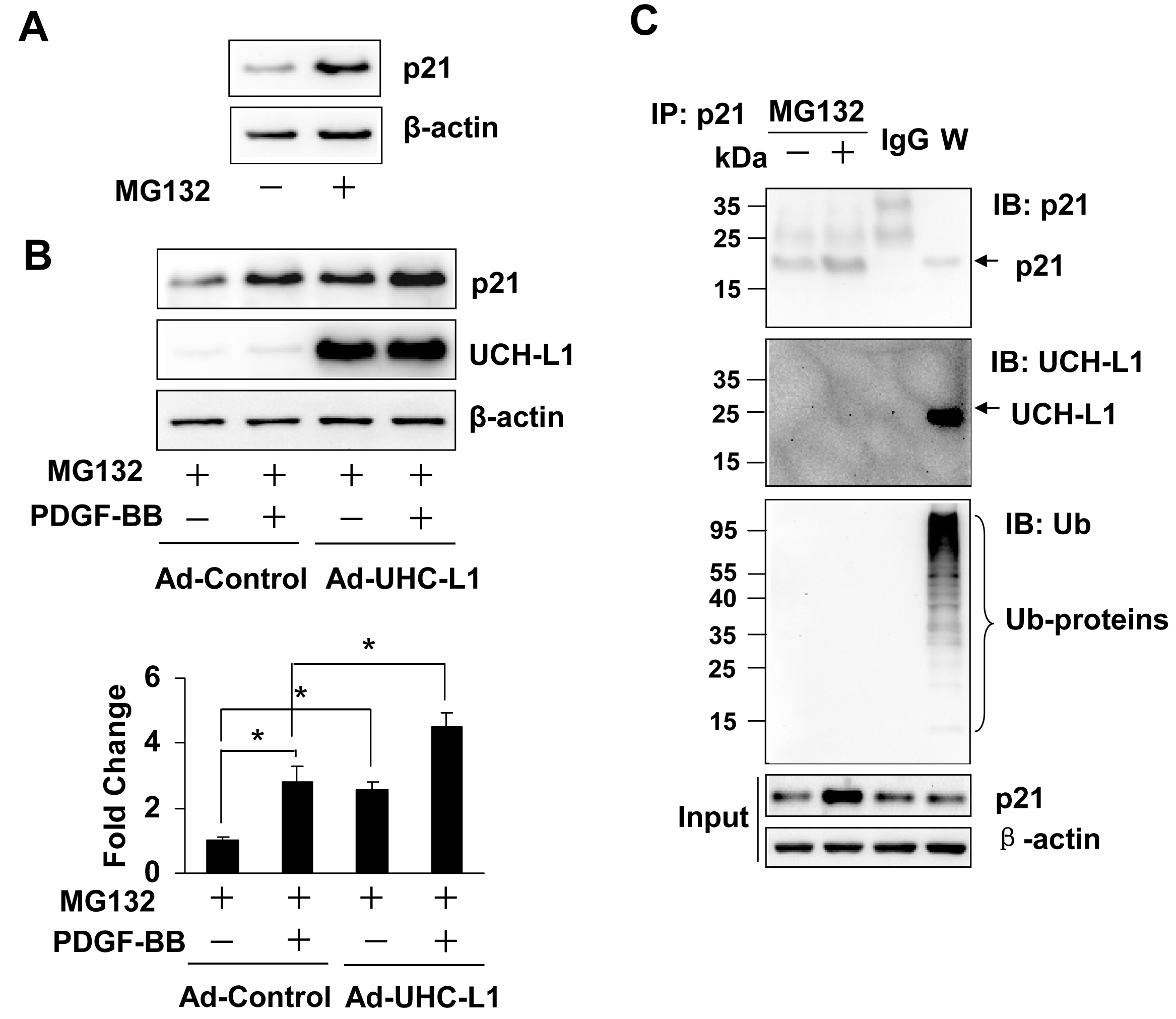

Supplement: Figure S7 — Effect of adenoviral UCH-L1 overexpression on ubiquitin proteasome system (UPS)-mediated degradation of p21 proteins in cardiac fibroblasts. A. Effect of MG132 on p21 protein expression in rat neonatal cardiac fibroblasts. Quiescent cells were treated with or without MG132 (0.5 µM) for 24 h. B. Effect of adenoviral UCH-L1 overexpression on PDGF-induced upregulation of p21 in the presence of MG132. Quiescent cells infected with Ad-control or Ad-UCH-L1 were treated with or without PDGF-BB (20 ng/ml) in the presence of MG132 (0.5 µM) for 24 h. Upper panel: representatives of immunoblotting. Lower panel: quantitatively densitometric analysis of protein expression. Data is presented as fold change of ratio of target protein to internal control β-actin relative to the Ad-Control PDGF-BB (-). n = 4, *p<0.05. C. Effect of MG132 on p21 protein ubiquitination as well as interaction of UCH-L1 and p21 proteins in rat neonatal cardiac fibroblasts. Quiescent cells were treated with or without MG132 (0.5 µM) for 24 h. W, whole cell lysates; IP, immunoprecitated; IB, immunoblotted. Input, 10 µg of whole cell lysates subjected to IB. All results are representatives of at least 4 separated experiments. (TIF) [file pone.0094658.s007.tif]

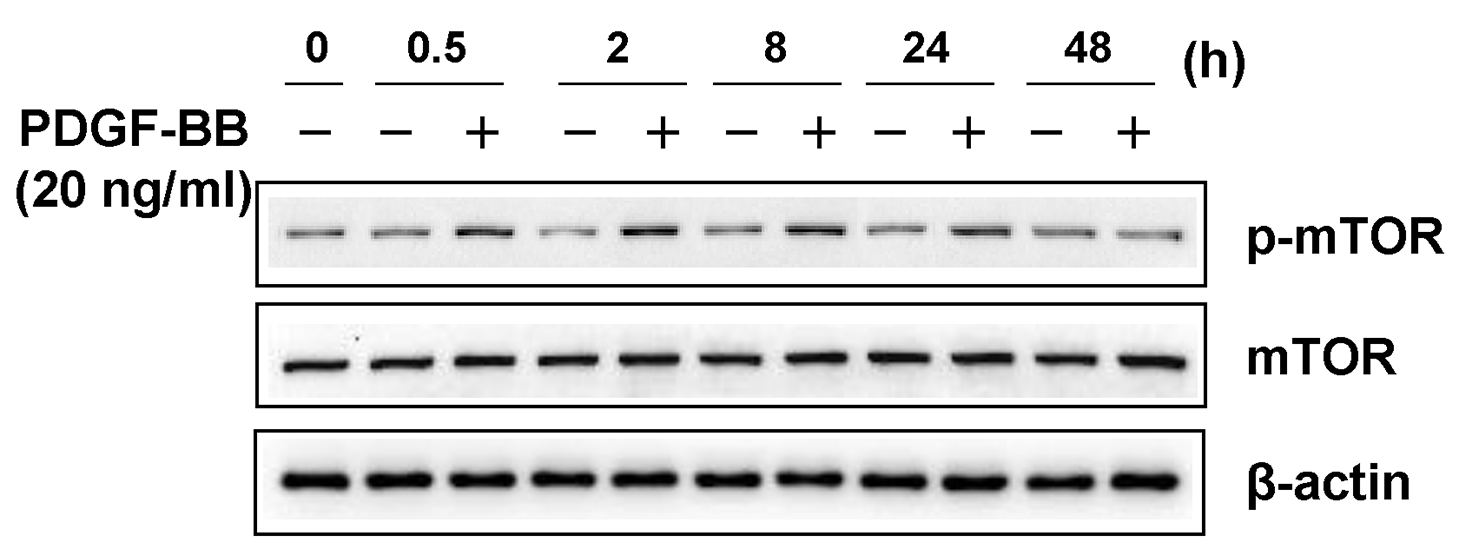

Supplement: Figure S8 — Effect of PDGF-BB on mTOR activity. Rat neonatal cardiac fibroblasts (passage 2) at 90% confluent status were cultured with serum free DMEM for 24 h to induce a quiescent status, and then stimulated with PDGF-BB (20 ng/ml) for different time periods as indicated. The cell lysates were subjected to Western blot analysis. Results are representatives of 4 separated experiments. (TIF) [file pone.0094658.s008.tif]

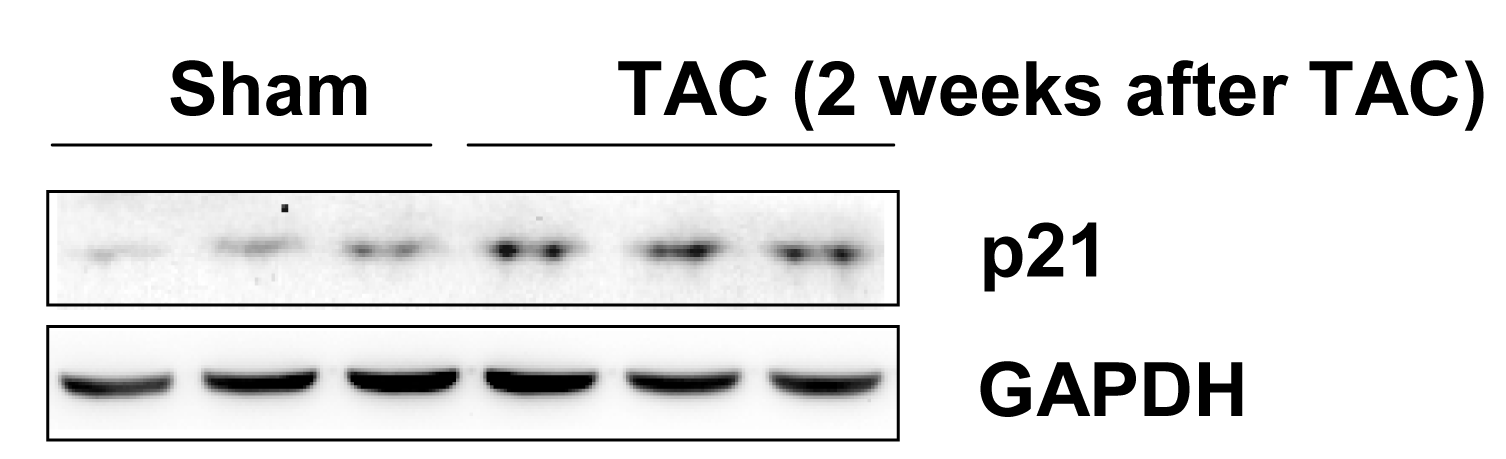

Supplement: Figure S9 — Western blot analysis of p21 expression in the left ventricles of mice 2 weeks after sham and TAC. n = 3. The results are representatives of 4 separated experiments. (TIF) [file pone.0094658.s009.tif]
