# Supplementary material for: Ubiquitin Carboxyl Terminal Hydrolyase L1 -Suppressed Autophagic Degradation of p21WAF1/Cip1 as a Novel Feedback Mechanism in the Control of Cardiac Fibroblast Proliferation
Source: PLoS One. 2014 Apr 14;9(4):e94658. doi: 10.1371/journal.pone.0094658 (PMC3986084; doi:10.1371/journal.pone.0094658)
Supplement: Methods S1 — Supporting methods. (DOC) [file pone.0094658.s010.doc]

Supporting Information

Methods

Immunohistochemistry

Hearts were cannulated via the left ventricular apex, cleared by perfusion with PBS at 90 mmHg, fixed by perfusion with 10% formalin, and embedded in paraffin. Paraffin sections were prepared (5 µm, Leica RM2030, rotary microtome) and stored at room temperature until staining. Immunohistochemistry was performed using an EnVision Detection Kit (GK500705, DAKO, Denmark) according to the manufacturer's instructions. Briefly, paraffin embedded sections were deparafinized, rehydrated, microwaved in 0.01 mol/L citrate buffer for 20 minutes for antigen retrieval, and incubated in 3% hydrogen peroxide for 15 minutes to quench endogenous peroxidase. The sections were further incubated in 5% bovine serum albumin (BSA) diluted in normal goat serum (Zhongshan, China) for 1 hour at room temperature (RT), then incubated with a rabbit anti-UCH-L1 polyclonal antibody (AB1761, Millipore) overnight at 4°C, followed with a ChemMate EnVision+/HRP secondary antibody for 30 minutes at RT. Thereafter, the sections were developed using a DAB Substrate for 30 seconds and then counterstained with hematoxylin. Images were acquired using a microscope (Nikon Eclipse 80i; Nikon Inc., Melville, NY).

Immunofluorescent staining was performed according to a standard protocol provided by Santa Cruz Biotechnology, Inc.. Ki67, a biomarker of cell proliferation, was stained with a rabbit anti-Ki67 antibody (ab15580, Abcam). Cardiac fibroblasts were stained with a mouse anti-Vimentin antibody (V2258, Sigma-Aldrich). Nuclei were labeled with 4’,6-diamidino-2- phenylindole (DAPI; D9542, Sigma-Aldrich).

Cell Culture and Adenovirus Infection

Rat neonatal cardiac fibroblasts were isolated and cultured as previously described . Briefly, the hearts from 2- to 3-day old Wistar rats were finely minced and digested with type II collagenase (120 units/ml; Worthington Biochemical Corp., Lakewood, NJ). Dispersed cells were placed in culture flask for 90 minutes at 37°C in a CO_2_ incubator. During this time, only the fibroblasts became attached to the culture flask. The fibroblasts were cultured in high glucose DMEM supplemented with 10% fetal bovine serum (FBS) (Gibco), penicillin (100 U/ml), and streptomycin (100 mg/ml). In addition, adult cardiac fibroblasts were isolated from male New Zealand (NZ) while rabbits at age of 6 months. The rabbits were euthanized by overdose intraperitoneal injection of pentobarbital sodium and then hearts were excised, minced, and washed in phosphate-buffered saline (PBS). The tissue was digested at 37°C with a mixture of trypsin (0.125%, Invitrogen) and type II collagenase (100 units/ml; Worthington Biochemical Corp.) for 10 min. Isolated cells were pelleted at the end of several 10-min digestion periods, plated on culture flask in DMEM containing 20% FBS, and incubated for 1 h at 37°C in a CO_2_ incubator. Thereafter, the unattached cells were discarded and attached cells were grown in DMEM with 10% FBS, penicillin (100 U/ml), and streptomycin (100 mg/ml). The purity of the fibroblasts was determined by the staining of a fibroblast marker vimentin using anti-Vimentin antibody (V2258, Sigma-Aldrich). Over 95% of the cultured cells were vimentin positive. Cardiac fibroblasts at passage 2 or 3 were used for the experiments.

Sub-confluent (70-80%) rat neonatal cardiac fibroblasts were infected with adenovirus of control (Ad-GFP) or human UCH-L1 tagged with GFP (Ad-hUCH-L1) (Invitrogen) in serum free DMEM for 48 h and followed with various treatments as indicated. Adenoviral over-expression of hUCH-L1 (0 - 100 pfu/cell) resulted in dose-dependent increases in UCH-L1 protein expression (Supplementary figure 1), without any apparent cytotoxic effects in cardiac fibroblasts (data not shown). Accordingly, we used adenoviral over-expression of UCH-L1 at a dose of 50 pfu/cell in the following experiments.

CCK-8 Assay

Rat cardiac fibroblasts (passage 2) were seeded in 96-well plate with 5000 cells/well, and were cultured in serum free DMEM for 24 h to induce a quiescent status. Then, cells were stimulated with or without PDGF-AA (50 ng/ml, P3076, Sigma-Aldrich), PDGF-BB (20 ng/ml, Sigma-Aldrich), PDGF-CC (50 ng/ml, SRP3139, Sigma-Aldrich), or PDGF-DD (50 ng/ml, 1159-SB, R＆D) for 48 hours. Cell proliferation was assessed by a Cell Counting Kit-8 (CCK-8, Dojindo Molecular Technologies, Gaithersburg, MD). Briefly, after stimulation, the CCK-8 solution was added to the culture medium, and the cultures were incubated for 1 hour at 37°C in humidified 95% air and 5% CO2. The absorbance was measured at 450 nm using a Microplate Reader (Bio-Rad, Hercules, CA).

Cell Counting

Rat cardiac fibroblasts (passage 2) were seeded in 6-well plates and cultured in DMEM supplemented with 10% FBS, penicillin (100 U/ml) and streptomycin (100 mg/ml). When cells reached to 50-60% confluent, cells were infected with adenovirus of UCH-L1 or GFP (50 pfu/cell) in serum free DMEM for 48 h, and then stimulated with PDGF-BB (20 ng/ml, Sigma-Aldrich) for additional 48 hours. Cells were trypsinized and counted using TC10 Automated Cell Counter (Bio-Rad, Hercules, CA).

Reverse Transcription-Polymerase Chain Reaction (RT-PCR) and Quantitative Real Time PCR (qPCR)

Total RNA from the left ventricles or cardiac fibroblasts was extracted using TRIzol (15596-018, Invitrogen) following the standard protocol, and reverse transcription reactions (RevertAid First Strand cDNA Synthesis Kit, K1622, Fermentas) were performed with 2 µg of total RNA. Quantitative real time PCR (Q-PCR) was carried out using the Bio-Rad CFX9 Real-Time System (Bio-Rad, Hercules, CA). Expression levels of target genes were normalized by concurrent measurement of glyceraldehydes-3-phosphate dehydrogenase (GAPDH) or β-actin mRNA levels. Primers that were used for Q-PCR are as follows: forward primer (5’-CCCCGAGATGCTGAACAAAGT-3’) and reverse primer (5’-ATGGTCTGCTTCATGAAGTA-3’) were used for PCR amplification of rat UCH-L1 (NM_017237.3) and mouse UCH-L1 ([NM_011670.2](http://www.ncbi.nlm.nih.gov/nucleotide/188219613?report=genbank&log$=nucltop&blast_rank=60&RID=XRM6SNXU01R)). Forward primer (5’-TCCGATCCTGGTGATGTCC-3’) and reverse primer (5’-CGAACACGCTCCCAGACGT-3’) were used for PCR amplification of rat p21 (NM_080782.3). Forward primer (5’-ACCACAGTCCATGCCATCAC-3’) and reverse primer (5’-TCCACCACCCTGTTGCTGTA-3’) were used for PCR amplification of mouse GAPDH (NM_008084.2). Forward primer (5’-CGTTGACATCCGTAAAGACC-3’) and reverse primer (5’-TAGAGCCACCAATCCACACA-3’) were used for PCR amplification of rat β-actin (NM_031144.3).

Western Blot Analysis

Cell lysate preparation and Western blot were performed as previously described . The primary antibodies of anti-UCH-L1 (AB1761), anti-UCH-L1 (ab8189), and anti-β-actin (TA-09) were purchased from Millipore, Abcam, and Zhongshan in China, respectively. Anti-phospho (Thr202/Tyr204)-ERK1/2 (#9101, ), anti-ERK1/2 (#4695), anti-phospho (Thr180/Tyr182)-p38 (#4631), anti-p38 (#4631), anti-phospho (Thr183/Tyr185)-JNK (#4668), anti-JNK (#9258), anti-phospho (Ser473)-Akt (#4058), anti-Akt (#9272), anti-phospho (Ser727)-Stat3 (#9134), anti-Stat3 (#4904), anti-phospho (Ser21/9)-GSK-3α/β (#5676), anti- GSK-3α/β (#5676), anti-phospho (Ser2448)-mTOR (#5536), anti-mTOR (#2983), and anti-LC3B (#2775) were purchased from Cell Signaling Technology. Anti-p21 (sc-397), anti-p27 (sc-528), anti-Cdk2 (sc-163), anti-Cdk4 (sc-260), anti-cyclin D1 (sc-718), anti-cyclin E (sc-481), and anti-GAPDH (sc-25778) were purchased from Santa Cruz. Bands were visualized with Immobilon Western Chemiluminescent HRP substrate (WBKLS0100, Millipore). Densitometric analysis was performed using AlphaView SA image software (Cell Biosciences).

Immunoprecipitation

Cell lysate preparation and Western blot were performed as previously described . Briefly, insoluble material was removed by centrifugation at 14,000 *g* for 15 min at 4°C. Protein concentration was measured in the cleared supernatant using BCA method. Then 40 µl protein A+G Agrose (P2012, Beyotime, China) and 1µg normal rabbit IgG per 1 ml of cell lysates were added to same amount of protein, and incubated at 4°C overnight with gentle rotating. The precleared lysates were incubated with 1 µg rabbit anti-p21 antibody (sc-397, Santa Cruz) with constant rotating at 4°C overnight, and then were incubated with protein A+G Agrose for 2 hours. The immunoprecipitates were washed 5 times with cell lysis buffer, then, the bound proteins were denatured with 40 µl 2×sample buffer. 20 µl samples for each well were subjected to SDS-PAGE.

References

1. Li J, Ichikawa T, Villacorta L, Janicki JS, Brower GL, et al. (2009) Nrf2 protects against maladaptive cardiac responses to hemodynamic stress. Arterioscler Thromb Vasc Biol 29: 1843-1850.

2. Cui TX, Nakagami H, Nahmias C, Shiuchi T, Takeda-Matsubara Y, et al. (2002) Angiotensin II subtype 2 receptor activation inhibits insulin-induced phosphoinositide 3-kinase and Akt and induces apoptosis in PC12W cells. Mol Endocrinol 16: 2113-2123.
